# PBSIM3: a simulator for all types of PacBio and ONT long reads

**DOI:** 10.1093/nargab/lqac092

**Published:** 2022-12-01

**Authors:** Yukiteru Ono, Michiaki Hamada, Kiyoshi Asai

**Affiliations:** Department of Computational Biology and Medical Sciences, Graduate School of Frontier Sciences, University of Tokyo, 5-1-5 Kashiwanoha, Kashiwa 277-8561, Japan; Department of Electrical Engineering and Bioscience, Faculty of Science and Engineering, Waseda University, 55N-06-10, 3-4-1, Okubo, Shinjuku-ku, Tokyo 169-8555, Japan; Computational Bio Big-Data Open Innovation Laboratory (CBBD-OIL), National Institute of Advanced Industrial Science and Technology (AIST), 63-520, 3-4-1, Okubo, Shinjuku-ku, Tokyo 169-8555, Japan; Institute for Medical-Oriented Structural Biology, Waseda University, 2-2, Wakamatsu-cho, Shinjuku-ku, Tokyo 162-8480, Japan; Graduate School of Medicine, Nippon Medical School, 1-1-5, Sendagi, Bunkyo-ku, Tokyo, 113-8602, Japan; Department of Computational Biology and Medical Sciences, Graduate School of Frontier Sciences, University of Tokyo, 5-1-5 Kashiwanoha, Kashiwa 277-8561, Japan; Artificial Intelligence Research Center (AIRC), National Institute of Advanced Industrial Science and Technology (AIST), 2-3-26, Aomi, Koto-ku, 135-0064 Tokyo, Japan

## Abstract

Long-read sequencers, such as Pacific Biosciences (PacBio) and Oxford Nanopore Technologies (ONT) sequencers, have improved their read length and accuracy, thereby opening up unprecedented research. Many tools and algorithms have been developed to analyze long reads, and rapid progress in PacBio and ONT has further accelerated their development. Together with the development of high-throughput sequencing technologies and their analysis tools, many read simulators have been developed and effectively utilized. PBSIM is one of the popular long-read simulators. In this study, we developed PBSIM3 with three new functions: error models for long reads, multi-pass sequencing for high-fidelity read simulation and transcriptome sequencing simulation. Therefore, PBSIM3 is now able to meet a wide range of long-read simulation requirements.

## INTRODUCTION

Long reads, such as Pacific Biosciences (PacBio) and Oxford Nanopore Technologies (ONT), have made it possible to detect structural variants, phase haplotypes and assemble genomes at high resolution (1,2). Typical read lengths range from 10 to 50 kb for PacBio continuous long reads (CLRs), from 12 to 24 kb for PacBio high-fidelity (HiFi) reads and from 10 to 100 kb for ONT reads. The ONT ultralong reads exceed 100 kb. The long reads span most repeat structures of the genomes, and ultralong reads, in particular, enable highly continuous genome assembly. In the analysis of variable number tandem repeats (VNTRs), the long reads can directly determine the total length of the VNTRs, which enables more accurate studies (3). Transcriptome sequencing (TS) using long reads has enabled the detection of differences in exon composition and the splicing of complex RNAs, which could improve the identification accuracy of isoforms (4,5). Long reads have a much higher error rate (typically 10–15%) than short reads (typically 0.1%), but their weaknesses have been quickly overcome by the development of tools and algorithms. In genome assembly, hybrid error correction has been performed using high-quality short reads to achieve a genome assembly with an error rate of 0.1% or less (6). After this, similar or improved accuracy was achieved through error correction using only long reads (7). PacBio’s recent HiFi reads achieved an accuracy of 99.9% through multi-pass sequencing, and long and accurate reads are expected to enable unprecedented analyses (8,9).

Many tools and algorithms have been developed to analyze long reads, and rapid progress in PacBio and ONT technologies has further accelerated their development (10–12). Fast algorithms for read mapping tools have been developed for short reads, and various other tools such as variant callers have also been developed. For the long reads that followed the short reads, tools and algorithms for utilizing long reads have been developed in a wide range of fields to deal with their high error rate. Together with the development of high-throughput sequencing technologies and their analytical tools, many read simulators have been developed and effectively utilized (13,14). In the development of tools and algorithms for long-read sequencers, it is generally difficult to evaluate them using real data. This is because real data that meet the necessary conditions cannot always be prepared. Additionally, the true error information of real data is not easy to obtain. Therefore, simulators that generate reads with error information, such as alignments between reads and reference sequences, are useful for evaluating new tools and algorithms.

PBSIM (15) is an early developed PacBio read simulator. PBSIM simulates reads based on the random error of PacBio reads. PacBio sequencing errors are considered stochastic (16); thus, PBSIM randomly picks a quality score for each position in a simulated read from a frequency table of quality scores and determines the presence and type of error according to both the quality score and the user-specified error ratio (substitution:insertion:deletion rates). The nucleotide sequences are ignored in this error introduction process. However, it has been reported that PacBio reads also have error biases (or context-specific errors), although they are weaker biases than that of short reads (17,18). To simulate the error biases of PacBio reads, many simulators have been developed to determine error patterns from alignments between long reads and their reference genomes. If the reference genome is identical to the genome from which the reads are sequenced, and if we can obtain accurate alignments, we should have a complete picture of how errors occur during the sequencing process. Using the alignments, LongLSLND (19), PaSS (20) and Badread (21) constructed context-dependent error models to simulate PacBio reads, and SNaReSim (22) also simulated ONT reads using a similar approach. It should, however, be noted that a perfect alignment is not yet possible and the algorithm and performance of the alignment tool will affect the estimated error models, producing additional bias (20).

To perform accurate genome assembly and variant calling using long reads, it is important to understand their error biases. To develop tools and algorithms that analyze long reads, data with accurately simulated error biases are required. Context-dependent error models can accurately capture the error biases, but differences in error biases between sample data were observed. In contrast, randomness-based PBSIM cannot simulate context-dependent error biases, but the simplicity thereof makes it possible to simulate typical long reads. PBSIM2 (the next version of PBSIM) (23) also did not implement a context-dependent error model but introduced a simulation of the nonuniformity of quality scores, which we consider to be just as important.

PBSIM3 implements the following three functions: The first is the hidden Markov model (HMM) for errors (called the ‘error model’), which simulates long reads, similar to the quality score model of PBSIM2. The second is a multi-pass sequencing simulation for PacBio Sequel HiFi reads. The third is a simulation of TS. PBSIM2 can simulate whole-genome sequencing (WGS) of PacBio RS II CLR and ONT reads. PBSIM3 can simulate WGS and TS of PacBio RS II CLR, PacBio Sequel CLR, PacBio Sequel HiFi and ONT reads. PBSIM3 can now meet a wide range of long-read simulation requirements.

## MATERIALS AND METHODS

### Datasets of real long reads

We used two PacBio RS II CLR, three PacBio Sequel CLR and four ONT real datasets (24) to develop the error models (Supplementary Table S1).

We also used two PacBio Sequel HiFi real datasets to characterize the HiFi reads (25) (Supplementary Table S1). HiFi reads have a median accuracy exceeding 99.9% (1,9). To obtain reliable error statistics of such highly accurate HiFi reads from alignments between the reads and their reference genomes, the following errors must be removed: (i) errors due to the difference in the cell line between reads and their reference genome, such errors or mutations are registered in public databases such as the Genome in a Bottle Consortium (GIAB) (26); (ii) the difference between haplotypes, to overcome this, a haplotype-resolved assembly and an accurate estimate of which haplotype it came from are required; (iii) errors due to incorrect assembly of repetitive sequences, which are difficult to accurately assemble. The *Homo sapiens* CHM13 cell line has an almost homozygous genome. The genome of this cell line was assembled by combining PacBio HiFi reads and ONT ultralong reads (25). Ultralong reads can span most repetitive sequences in the genome, which results in high-resolution repetitive sequence assembly. Therefore, *H. sapiens* CHM13 reference genomes and reads are expected to be largely unaffected by the errors described above. For other HiFi data, *Escherichia coli* is a haploid, and the reference genome and reads used here were sequenced from the same strain, *E. coli* K12. In addition, long reads span most repeats of the bacterial genome (27). Therefore, the sample should be unaffected.

To characterize TS using long reads, we used two datasets of PacBio Iso-seq HiFi reads, three datasets of ONT direct RNA reads and two datasets of ONT direct complementary DNA (cDNA) reads (28,29) (Supplementary Table S2).

### Read alignment

To characterize the long reads of WGS, we conducted local alignments of real reads to their reference genomes, calculated error rates and examined error biases from the alignment results (Supplementary Table S3). These local alignments were executed using LAST version 1111 (30), and the alignments were filtered using last-split (31). lastal was executed using the score matrix trained by last-train (32). The parameter settings are presented in Supplementary Table S4.

To characterize the long reads of TS, we conducted local alignments of real reads to the human transcriptome (Ensembl GRCh38 release-104 cDNA and ncRNA (33)) (Supplementary Table S5). To accurately examine the sequencing bias of the transcript, we only used alignments with >95% read coverage. If a read mapped onto multiple isoforms with the same score, the isoforms were treated equally. These local alignments were executed using Minimap2, version 2.17-r941 (34). The parameter settings are presented in Supplementary Table S4.

To evaluate the simulation performance, we also conducted local alignments of simulated reads, as described above, and compared their characteristics with those of real reads.

### Generative model for errors

To construct a generative model for errors, we used an HMM that generates observed data from hidden states that follow the Markov model. The error model was built using FIC-HMM (HMM with factorized information criteria (35)) in the same manner as building the quality score model of PBSIM2 (23). The training data of FIC-HMM are alignments between long reads and their reference genomes. By converting the match, substitution, insertion and deletion on the alignment to 0, 1, 2, 3, respectively, we created sequences of numbers and used them as training data. By using a more accurate alignment as training data, a better model can be constructed. However, there are several causes of uncertainties in alignments, such as merging an insertion and its adjacent deletion into a match or substitution, or the inability to determine the true locations of insertions and deletions (INDELs) within homopolymers. In this study, INDELs in a homopolymer were randomly rearranged to reduce the bias caused by the habits of the aligner. In our HMM, the emission probability distributions from each hidden state are provided by a categorical distribution whose output is a match or error type. It should be emphasized that the parameters in the categorical distribution with hidden states differ from each other. In a conventional HMM, the number of hidden states must be provided beforehand. This method is theoretically sound, enabling us to train not only the parameters in the HMM but also the number of hidden states (36). In this study, we adopted a model whose (lower bound of) FIC is maximum among five trials with different initial parameters because FIC-HMM affects local optimal solutions in their training. The models were trained for individual read accuracy of each sample dataset (e.g. for 80% accuracy, training data comprised a read group with an accuracy of 79.5–80.4%). For read accuracy with insufficient training data, constant-quality scores that matched the accuracy were used.

### Execution of simulators

We conducted various simulations using PBSIM3 and evaluated the simulated reads from various aspects. The parameter settings are presented in Supplementary Table S4. In the multi-pass sequencing simulation, the parameter ‘–pass-num*number of passes*’ was specified. Read length, error rate and error ratio were adjusted to the values of the real reads. The error ratio could be specified for the quality score model but not for the error model. The error ratio could not be changed as it was built into the error model.

We also conducted simulations using Badread version v0.2.0 and NanoSim version v3.0.0 (37) for comparison with PBSIM3. Although both simulators offer functions for building an error model for each real dataset, we used their built-in models.

### Nonuniformity of error

**Figure 1.**
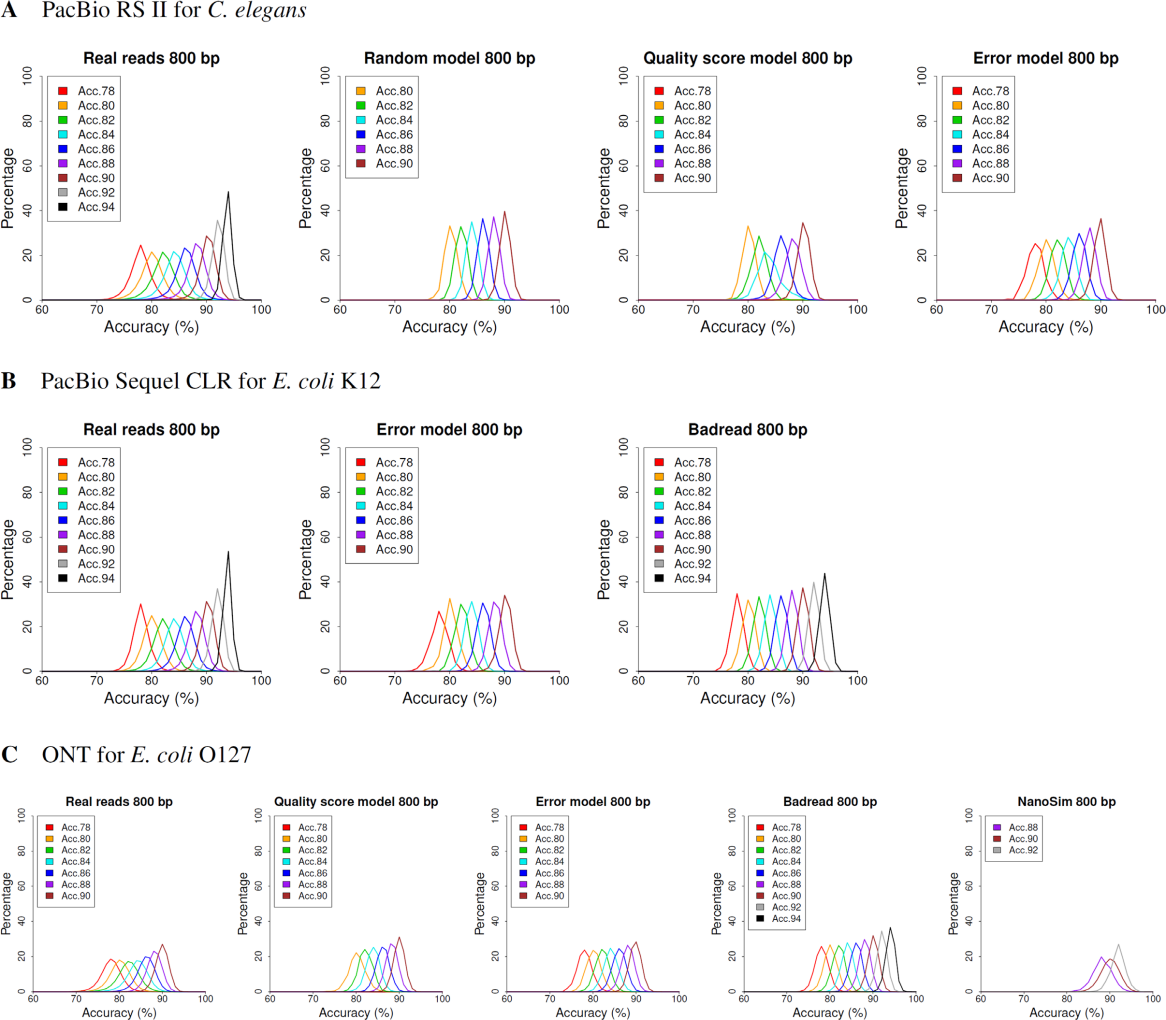
Nonuniformity of errors for real and simulated reads. After grouping reads by their accuracy, they were segmented into 800 bp disjoint intervals, and accuracy of each interval was computed from alignments between the reads and their reference genomes. Each graph shows the distribution of the averaged accuracy of 800 bp intervals, where the color of the plotted lines represents read groups (e.g. Acc.78 refers to a read group with an accuracy of 77.5–78.4%). The random model randomly generates errors according to an error rate and error ratio.

Long reads have a regional bias of error distribution within the reads, and very low-quality regions are sometimes observed (e.g. Myers’ report, https://dazzlerblog.wordpress.com/2015/11/06/). One of the main causes is the nonuniformity of errors; therefore, we developed the quality score models of PBSIM2 and showed that the models can accurately simulate the nonuniformity of quality scores but not the errors (23). In this study, we evaluated the error model of PBSIM3 to simulate the nonuniformity of errors (Figure 1).

To measure the similarity of the nonuniformity of errors, we used the Kullback–Leibler (KL) divergence between the real and simulated reads (Supplementary Figure S4). For *P* (real distribution) and *Q* (simulated distribution), the KL divergence from *Q* to *P* is defined as

\begin{equation*} D_{{\rm KL}}(P||Q)=\sum _i P(i)\log _2\frac{P(i)}{Q(i)}. \end{equation*}

### Error bias in homopolymers

To measure the error bias in homopolymers, the homopolymer length of each site in the genome was first determined. If a site is in a region where *N* identical bases are continuous, it is designated as *N*. Then, for each of the homopolymer lengths, the number of errors was counted. The number of sites with homopolymer lengths of 1–3 was overwhelmingly large, and most errors occured at these sites. However, in terms of error rate, it was found in HiFi and ONT reads that the longer the homopolymer length, the higher the error rate (Supplementary Figures S6–S9).

### Generation of consensus reads (HiFi read)

Multi-pass sequencing of subreads was simulated using PBSIM3. Using the PacBio BAM format data generated by PBSIM3 as an input, ccs (version 6.0.0, https://github.com/PacificBiosciences/ccs) was executed with default parameters to generate consensus reads (HiFi reads).

## RESULTS

### Simulation by a generative model of errors

**Figure 2.**
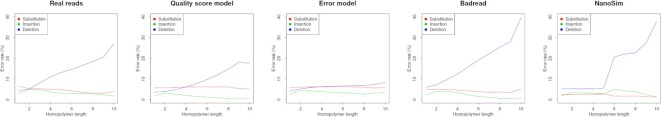
The error bias in homopolymers of ONT R10.3 reads for *E. coli* O127. If a site is contained in a genomic region where *N* identical bases are continuous, homopolymer length is designated as *N*. Then, for each of the homopolymer lengths, the number of errors was counted and the error rate calculated. ONT reads were simulated using the quality score model, error model, Badread and NanoSim. The error and quality score model were executed with the parameter ‘–hp-del-bias 6’. The error rates were calculated from alignments between the reads and their reference genomes.

The quality score model used in PBSIM2 simulates the quality scores and introduces errors based on the quality scores. This model does not distinguish between error types (substitution, insertion and deletion). So, it allows the users to simulate with any ratio of error types; however, the model cannot accurately simulate the characteristics of error types. K-mer profiles have been widely used to simulate the characteristics of error types. These profiles consist of error patterns and probabilities for each k-mer, and are created from the alignments between real reads and the reference sequences thereof. LongISLND (19), PaSS (20) and Badread (21) have adopted this approach. These tools learned real reads to create a model that included the k-mer profile and then simulated reads using the model. Nevertheless, PBSIM3 adopted FIC-HMM, which was also used in PBSIM2, without using k-mer profiles. Our error model is sequence independent, whereas the k-mer profile is sequence dependent. Several sequence-dependent errors in long reads have been reported. For example, in ONT, there are many INDELs in homopolymers, and the ratio of A-G/G-A in the substitution pattern is predominant (38,39). These errors are commonly observed in many ONT datasets, but there are other dataset-specific k-mer profile characteristics (data not shown). Therefore, a k-mer profile should be created for each dataset as many simulators do. We prioritized usability and took a sequence-independent approach that could be easily used for many reference genomes but added the option of introducing the homopolymer bias of deletion into the ONT reads. This is because the deletion bias is still observed in the R10.3 chemistry (Figure 2), even though the homopolymer bias has been improved in both the sequencer and basecaller (40).

Nonuniformity of errors was observed in the long reads. PBSIM2 makes it possible to simulate the nonuniformity of quality scores but not the errors by introducing a quality score model (23). Quality scores and errors are well correlated; therefore, it is likely that the quality score model can also simulate the nonuniformity of errors. In PBSIM3, we developed error models that directly simulate the errors and evaluated them for the simulation performance of the nonuniformity of errors, as well as the quality score models. Supplementary Figures S10 and S11 show the emission and transition probability matrices of the error models.

The simulation performance of each type of long read was evaluated in terms of the nonuniformity of errors, INDEL size and homopolymer bias. In all the simulations, the total number of bases in the simulated reads was approximately 100 million. The read length and error rate were the default values in the NanoSim simulation. In the other simulations, we set the average read length to 15 kb, the standard deviation to 15 kb and the error rate to 15%, in reference to the real read values in Supplementary Table S1. Most of the real read error rates obtained from the alignment were <15%, but the error rate of alignment tended to be lower than the true error rate (Supplementary Table S6). Therefore, the error rate was set slightly higher.

First, PacBio RS II CLR reads were simulated using the random, quality score and error models. The nonuniformity of errors was compared between the simulated and real reads. The ‘random model’ randomly generated errors according to the error rate and error ratio. The error ratio was built into the error model. For the other models, the error ratio was 6:55:39. In previous studies, the simulation of quality score nonuniformity with the quality score model showed a slightly worse performance at a low accuracy of 78–80% (23). Similarly, in the simulation of nonuniformity of errors, the performance of the quality score model was worse at a low accuracy of 82% or less (Figure 1A, Supplementary Figures S1 and S4A). In contrast, the error model exhibited a high performance for all accuracies. In the distribution of INDEL size, the quality score model can simulate real reads correctly, but the error model is slightly larger than real reads (Supplementary Figure S5A). No homopolymer bias was observed in RS II CLR reads (Supplementary Figure S6).

Second, the PacBio Sequel CLR reads were simulated using the error model and Badread, and the nonuniformity of errors was compared between these simulated and real reads. The error ratio was built into the error model and Badread. Badread is a simulator that uses a k-mer profile and shows high performance for all accuracies (Figure 1B; Supplementary Figures S2 and S4B). The error model also showed high performance and was comparable to Badread. In the distribution of INDEL size, the error model can simulate a real read correctly (Supplementary Figure S5B). No homopolymer bias was observed in Sequel CLR reads (Supplementary Figure S7).

Finally, the ONT reads were simulated with the quality score model, error model, Badread and NanoSim, and the nonuniformity of errors was compared between these simulated and real reads. The error ratio was built into the error model, Badread and NanoSim. The quality score model was 39:24:36. NanoSim uses an alignment-based trained model that does not use a k-mer profile. The PBSIM3 error model approach is similar to that of NanoSim except for the learning method. The quality score model, error model and Badread can accurately simulate the nonuniformity of errors in all accuracies (Figure 1C, Supplementary Figures S3 and S4C). NanoSim has a narrow range of accuracy for generated reads, which is different from that of real reads. In the distribution of INDEL size, the error model can simulate real reads correctly, but the quality score model is slightly smaller than real reads (Supplementary Figure S5C). ONT reads have a deletion homopolymer bias in which the longer homopolymer length results in a higher deletion rate (Figure 2 and Supplementary Figure S8). Badread can simulate the bias correctly, most likely because of the k-mer profile. NanoSim does not use the k-mer profile, but the parameter ‘-k 6’ allows the bias to be simulated. Both PBSIM3 models are sequence independent; therefore, homopolymer bias cannot be simulated, but the parameter ‘-hp-del-bias 6’ allows the deletion homopolymer bias to be simulated (Figure 2 and Supplementary Figure S8C). The parameter specifies a deletion rate at 10-mer, where the deletion rate at 1-mer is 1. The bias intensity from 1- to 10-mer is proportional to the length of the homopolymer. However, the error model can only slightly simulates the bias. In the quality score model, the deletion rate can be changed flexibly; however, in the error model, the error ratio is built into the model, so the change is limited. When simulating homopolymer bias, a quality score model should be used.

### Simulation of multi-pass sequencing

Compared with single-pass reads, there are only a few simulators for multi-pass reads. PBSIM uses the random model of errors to simulate multi-pass sequencing (15). However, PBSIM only resembles the read length and accuracy distribution and does not incorporate the characteristics of multi-pass sequencing. Another software, SimLoRD (41) implements an error model that incorporates the characteristics of multi-pass sequencing. HI.SIM (https:// github.com/thegenemyers/HI.SIM) and Sim-it (42) directly simulate HiFi reads using sequence-dependent k-mer models. We determined that PBSIM3 does not directly simulate HiFi reads but only simulates the generation of CLR reads by multi-pass sequencing. The output of PBSIM3 simulation was directly input into ccs software, which generated consensus reads. It is reasonable to expect that a better simulation of HiFi reads can be achieved by using the real consensus read generation process.

Similar to CLR reads generated by single-pass sequencing, CLR reads generated by multi-pass sequencing are error prone. The error-prone reads are processed using ccs software to generate HiFi reads with very few errors. The simulated HiFi read must have the same error characteristics as the real HiFi reads, namely error rate, error ratio and high error rate in homopolymers, which have been frequently reported (1,43). The error rates obtained from the real reads and genomic sequence alignment were 0.22 and 0.25% (Supplementary Table S3), which are consistent with the reported error rate (1). The substitution rate was particularly low and the insertion and deletion rates were similar.

PacBio Sequel CLR reads were simulated using the random, quality score and two error models. The number of passes was 10. Three CLR error rates were tested: 10%, 15% and 20%. The error ratio was 22:45:33 for all data. The length of all the reads was 15 kb. In single-pass sequencing, PBSIM3 uses a gamma distribution for read length, whereas in multi-pass sequencing, it uses a constant length.

**Figure 3.**
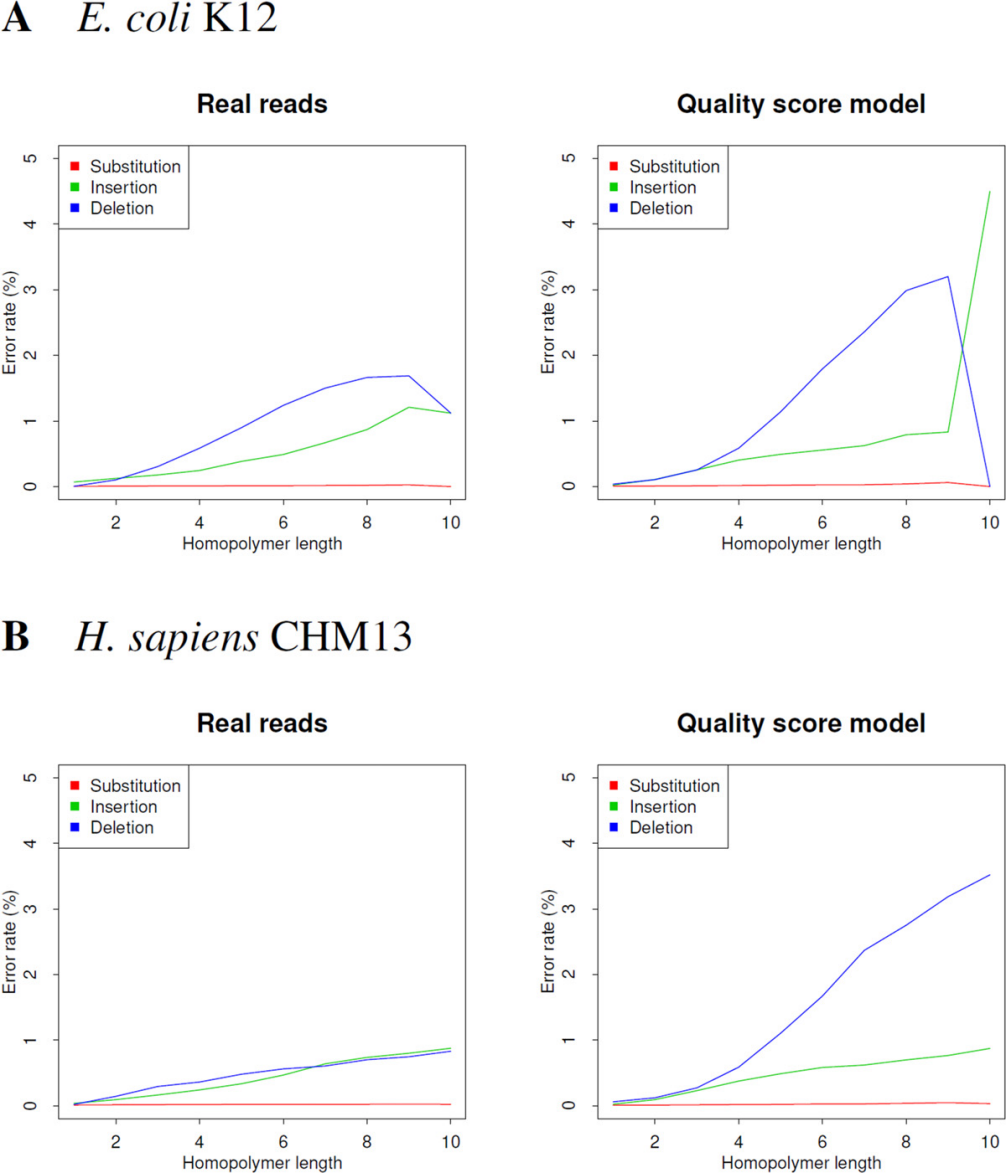
The error bias in homopolymers of PacBio HiFi reads. If a site is contained in a genomic region where *N* identical bases are continuous, homopolymer length is designated as *N*. Then, for each of the homopolymer lengths, the number of errors was counted and error rate was calculated. The simulated reads were generated by ccs software as consensus sequences from PacBio CLR reads simulated using the PBSIM3 quality score model. The CLR error rate was 15%.

Table 1 and Supplementary Table S7 show that when the number of passes is 10, CLR error rates of 10–15% can be used to simulate HiFi reads with an error rate similar to that of real HiFi reads. Compared to the error rate of real HiFi reads, the deletion rate was higher for all four simulation methods. In particular, the deletion rate was high in the two error models. For homopolymer error bias, simulated HiFi reads showed the same characteristics as real HiFi reads, with longer homopolymer lengths resulting in the higher error rates. However, the rate of increase in the deletion rate of simulated HiFi reads was greater than that of real HiFi reads (Figure 3 and Supplementary Figure S9). HiFi read simulation was tested with numbers of passes of 5, 15 and 20, using the quality score model (Supplementary Table S8). In the simulation with five passes, the error rate was high (0.99% for *H. sapiens* CHM13); notably, the yield rate of the consensus sequence from ccs was very low (23.73% and 98.27% after 10 passes). The number of passes must be changed depending on the CLR error rate, error ratio and error rate of the HiFi read selected.

**Table 1.** Simulation of HiFi reads (*H. sapiens* CHM13)

Real or simulators	CLR error	Sub.	Ins.	Del.	Total
	Rate	Rate	Rate	Rate	
Real reads		0.02%	0.10%	0.13%	0.25%
Random model	10%	0.01%	0.08%	0.11%	0.20%
	15%	0.02%	0.17%	0.24%	0.43%
	20%	0.03%	0.31%	0.45%	0.80%
Quality score model	10%	0.01%	0.06%	0.09%	0.16%
(RS II)	15%	0.01%	0.12%	0.20%	0.34%
	20%	0.03%	0.21%	0.37%	0.60%
Error model	10%	0.01%	0.12%	0.14%	0.27%
(RS II)	15%	0.01%	0.24%	0.39%	0.64%
	20%	0.02%	0.48%	0.83%	1.34%
Error model	10%	0.01%	0.06%	0.17%	0.24%
(Sequel)	15%	0.01%	0.12%	0.40%	0.53%
	20%	0.03%	0.17%	0.98%	1.18%

The reference genome is *H. sapiens* CHM13. Simulated HiFi reads were generated by ccs software as consensus sequences from simulated CLR reads. CLR reads were simulated with the random, quality score and error models. The error rates were obtained from the alignments between the reads and their reference genomes.

### Simulation of transcriptome sequencing

There are only a few simulators for the TS. simlady is a simulator for long-read RNA sequencing (44). simlady incorporates the process of 5’ RNA degradation into the simulation of TS. Trans-NanoSim is an extension of NanoSim to TS (45). From real reads and the alignment of real reads with their reference genome and transcriptome, Trans-NanoSim learns read characteristics such as read length distribution and error profile, while simultaneously creating the expression profile. Using these as inputs, Trans-NanoSim simulates the TS of ONT.

**Figure 4.**
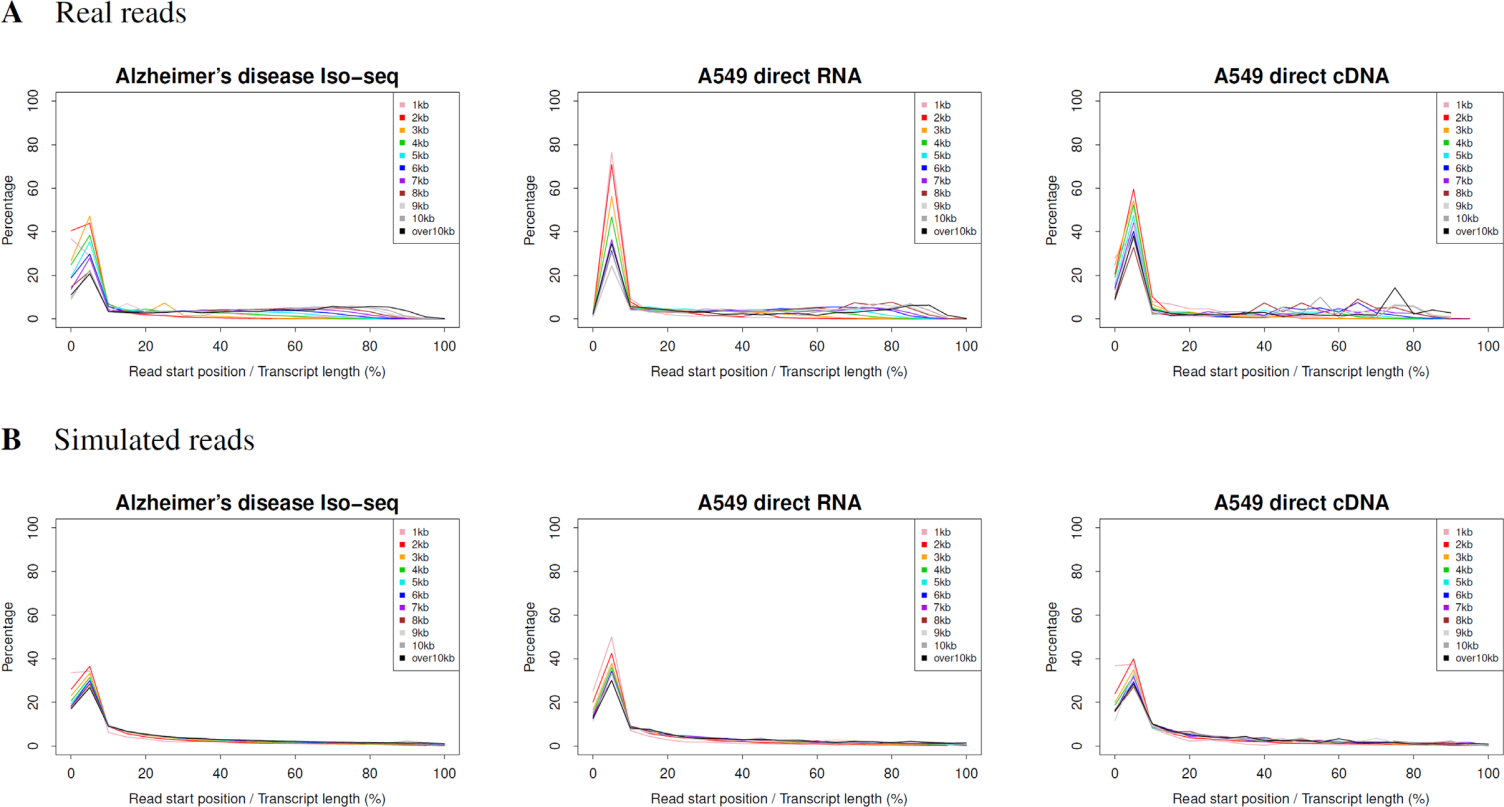
Read start positions on their template transcripts. Reads were grouped by 1 kb by their template length. Each graph shows the distribution of the read start positions, where colors of plotted lines represent read groups (e.g. 1 kb refers to a read group with their template length of 1–1000 bp). The horizontal axis indicates the position of the read start positions in the total length of their templates, which was calculated by dividing the read start position by the total length of the template; the graph is plotted in 5% increments, with the left edge of the graph showing the percentage of reads starting exactly at the 5’ end of the template. PacBio Iso-seq (CLR read), ONT direct RNA and ONT direct cDNA were simulated using the PBSIM3 quality score models. The Iso-seq (HiFi read) was generated by ccs software as consensus sequences from PBSIM3 outputs. The template transcript from which each read was most likely sequenced and the read start position was obtained from alignments between the reads and their reference transcriptomes.

**Figure 5.**
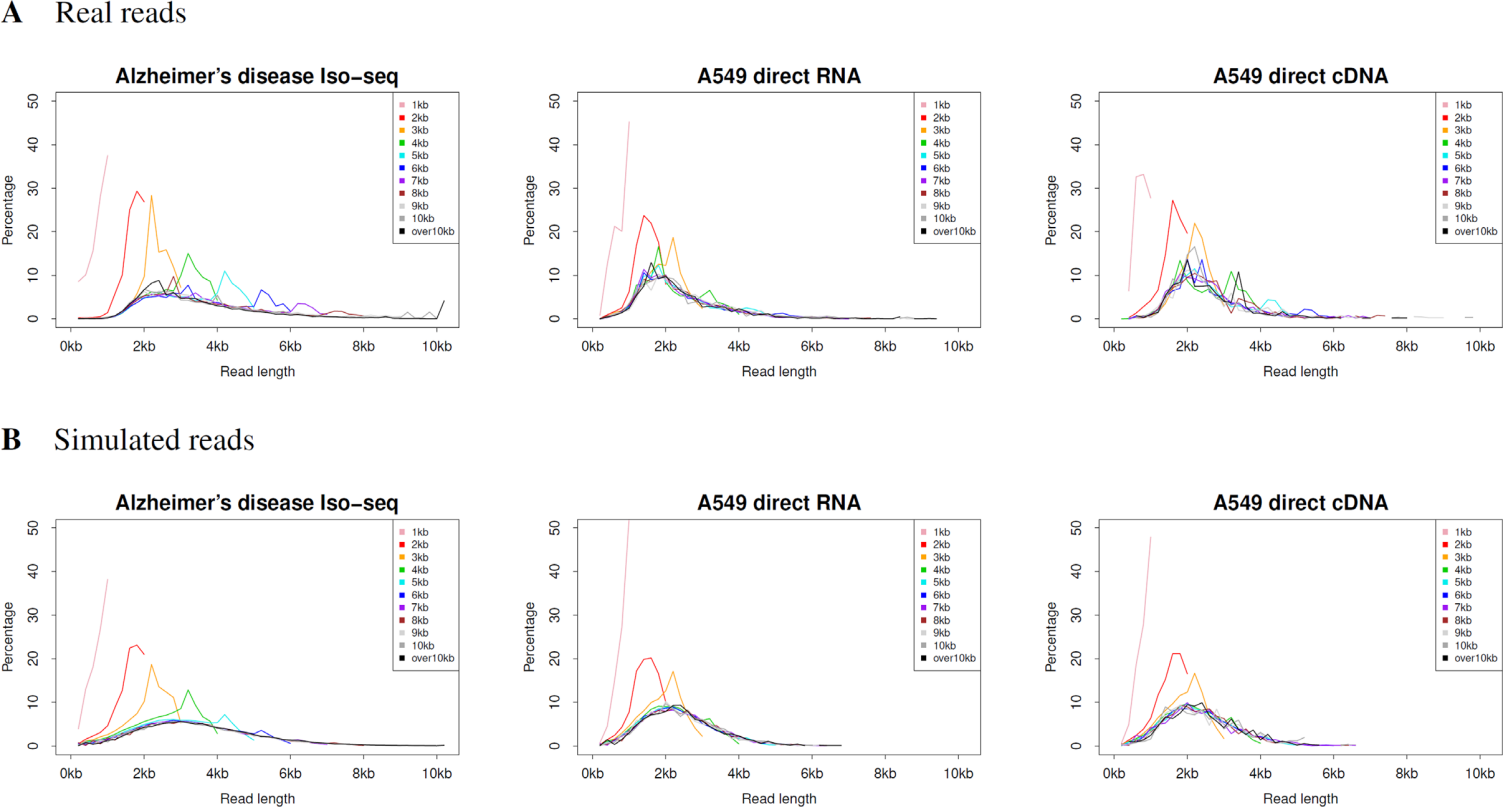
Read length distribution of TS. Reads were grouped by 1 kb by their template length. Each graph shows the distribution of the read length, where colors of plotted lines represent read groups (e.g. 1 kb refers to a read group with their template accuracy of 1–1000 bp). PacBio Iso-seq (CLR read), ONT direct RNA and ONT direct cDNA were simulated using the PBSIM3 quality score models. The Iso-seq (HiFi read) was generated by ccs software as consensus sequences from PBSIM3 outputs. The template transcript from which each read was most likely sequenced was obtained from alignments between the reads and their reference genomes.

Long-read TS can generate full-length isoform reads, which are not always possible because of cDNA or RNA degradation or fragmentation in sample preparation (46). The TS process from library preparation to sequencing is complicated and remains unknown. To simulate the TS process, we adopted a simple TS model that determines the position and length of reads on their template transcripts from which the reads were sequenced. We conducted alignments between real TS reads and their reference transcriptomes (Supplementary Table S5) and observed the read start positions on their template (Figure 4A) and read length distribution to template length (Figure 5A).

It has been reported that all current long-read TS are biased toward shorter reads (5) and that the read length is considerably shorter than that of the template (4). The real read length of the TS in Supplementary Table S2 and the read length distribution in Figure 5A are consistent with these previous reports. The read lengths of PacBio CLR and ONT in WGS follow a gamma distribution (23). By comparing the read length with the length of the templates, the read lengths still follow the gamma distribution, as long as the reads are sufficiently shorter than their templates (Figure 5A, and Supplementary Figures S13A, S15A and S17A). To simulate the read length distribution of TS, as with WGS, read lengths are determined according to the gamma distribution; reaching the 3’ end of the template terminates the simulation.

In terms of the position of the reads on their templates, it has reported that percentage of 5’ degraded transcript sequenced by Iso-seq was high (47), and that in ONT direct RNA sequencing, 5’ end truncations by the sequencer can make it difficult to define transcription start sites (4). ONT direct RNA sequencing proceeds from the 3’ end to the 5’ end of the template, leading to biased coverage toward the 3’ end of the template (12). The real read start positions on their templates in Figure 4A are consistent with previous reports. The mismatch at the 5’ ends between reads and templates is considered to be largely caused by cDNA or RNA degradation and fragmentation. We adopted Pareto distribution as the distribution of the read start positions.

We implemented the novel TS model in PBSIM3, where the user has to input the sequencing templates, that is, the transcript sequences and their expression profiles, into PBSIM3. This information is uploaded to PBSIM3 as a tab-delimited file. The file has a one-line-one-transcript format, the items have a transcript ID, and the number of expressions (sense), number of expressions (antisense) and nucleotide sequences are provided. PBSIM3 uses the TS model to generate a user-specified number of reads for each template transcript.

The quality score and error models for TS are the same as those for WGS. PacBio Iso-seq (CLR read), ONT direct RNA and ONT direct cDNA were simulated using the quality score model. Iso-seq (HiFi read) was generated using the ccs software as consensus sequences from the PBSIM3 outputs. The CLR and ONT error rates were set to 15% in all these simulations. For PacBio Iso-seq, the number of passes was 10 and the error ratio was 22:45:33. The average read length was 3.6 kb and the standard deviation was 1.6 kb. The read length in multi-pass sequencing is a constant value in WGS, but a gamma distribution is adopted in TS. For ONT direct RNA and cDNA, the error ratio was 39:24:36. The average read length was 2.4 kb and the standard deviation was 1 kb. As shown in Figures 4 and 5, and Supplementary Figures S12–S17, PBSIM3 simulated both the read start position and the length distribution well. However, the read length distributions of ONT direct RNA and cDNA were less accurate than that of PacBio Iso-seq. The TS model was developed based on the estimation of template isoforms; however, the isoform estimation is still challenging and in the process of improvement (48,49). We aim to improve the TS model using improved isoform estimation.

## DISCUSSION

With the introduction of the error model, PBSIM3 can now simulate the PacBio Sequel CLR reads. The error model performed better than the quality score model in the PacBio RS II CLR read simulation. However, the performance of the quality score model is satisfactory, and the error ratio can be changed as desired; therefore, it is more flexible than the error model.

To simulate PacBio Sequel HiFi read, we adopted the approach of combining PBSIM3 and ccs. This approach succeeded in generating reads with the characteristics of real reads. The advantage of this approach is that ccs version upgrades can easily be reflected in PBSIM3. The disadvantage is that it requires much greater computational resources than the PBSIM Sequel CLR or ONT read simulation. PBSIM3 generates a large number of CLR reads as intermediate data by multi-pass sequencing, and the computational resources of consensus sequence generation by ccs are added. The three models provided by PBSIM3 are capable of achieving the real error rate and error ratio of HiFi reads. The deletion rate tends to be simulated a bit higher, but one can simulate the desired HiFi reads by adjusting the CLR error rate, error ratio and number of passes. The combination of PBSIM3 and ccs can simulate the homopolymer bias observed in HiFi reads. Furthermore, HiFi reads are reported to have another bias at the end of dinucleotide and trinucleotide satellites, and HI.SIM includes this bias in its error model (https://github.com/thegenemyers/HI.SIM). Because the analysis of repetitive sequences with long reads has become increasingly popular (50), understanding the effect of repetitive sequences on long-read sequencing is necessary. In the future, we would like to incorporate knowledge on the effects of repetitive sequences into PBSIM3.

We created a TS model and implemented it in PBSIM3. In this model, the read start position on the templates and read length are determined from the template length. PBSIM3 can accurately simulate the real TS using this model. However, this model does not precisely simulate the real TS process but mimics the appearance of real TS reads. Simulation of the real TS process is a future challenge. Biases in RNA-seq library preparation have also been reported (51). This could affect long-read TS, and simulations of these factors should be incorporated into PBSIM in the future.

## CONCLUSION

In this study, PBSIM3 implements three new functions. First, we implemented error models for long reads. Error models were constructed using FIC-HMM as well as quality score models. The error models showed a high performance in the simulation of PacBio CLR and ONT reads. Second, multi-pass sequencing for HiFi read simulation was implemented. Using the CLR reads generated by PBSIM3 as input, ccs can simulate reads with the characteristics of real HiFi reads. Third, a TS simulation was implemented. We developed a novel TS model comprising two parts. The first part is the determination of where to set the read start position in a given template transcript. The second part is the determination of read length. PBSIM3 can accurately simulate TS for both the read start position and read length distribution. PBSIM3 can now meet a wide range of long-read simulation requirements.

## DATA AVAILABILITY

The source code for PBSIM3 is freely available at https://github.com/yukiteruono/pbsim3 and https://doi.org/10.5281/zenodo.7312115.

## Supplementary Material

lqac092_Supplemental_File

## References

[1] Wenger A.M., Peluso P., Rowell W.J., Chang P.C., Hall R.J., Concepcion G.T., Ebler J., Fungtammasan A., Kolesnikov A., Olson N.D. et al. Accurate circular consensus long-read sequencing improves variant detection and assembly of a human genome. Nat. Biotechnol. 2019; 37:1155–1162. 31406327 10.1038/s41587-019-0217-9 PMC6776680

[2] Bowden R., Davies R.W., Heger A., Pagnamenta A.T., de Cesare M., Oikkonen L.E., Parkes D., Freeman C., Dhalla F., Patel S.Y. et al. Sequencing of human genomes with nanopore. Nat. Commun. 2019; 10:1869. 31015479 10.1038/s41467-019-09637-5 PMC6478738

[3] Course M.M., Sulovari A., Gudsnuk K., Eichler E.E., Valdmanis P.N. Characterizing nucleotide variation and expansion dynamics in human-specific variable number tandem repeats. Genome Res. 2021; 31:1313–1324. 34244228 10.1101/gr.275560.121 PMC8327921

[4] Workman R.E., Tang A.D., Tang P.S., Jain M., Tyson J.R., Razaghi R., Zuzarte P.C., Gilpatrick T., Payne A., Quick J. et al. Nanopore native RNA sequencing of a human poly(A) transcriptome. Nat. Methods. 2019; 16:1297–1305. 31740818 10.1038/s41592-019-0617-2 PMC7768885

[5] Byrne A., Cole C., Volden R., Vollmers C. Realizing the potential of full-length transcriptome sequencing. Philos. Trans. R. Soc. Lond. B Biol. Sci. 2019; 374:20190097. 31587638 10.1098/rstb.2019.0097 PMC6792442

[6] Koren S., Schatz M.C., Walenz B.P., Martin J., Howard J.T., Ganapathy G., Wang Z., Rasko D.A., McCombie W.R., Jarvis E.D. et al. Hybrid error correction and *de novo* assembly of single-molecule sequencing reads. Nat. Biotechnol. 2012; 30:693–700. 22750884 10.1038/nbt.2280 PMC3707490

[7] Chin C.S., Alexander D.H., Marks P., Klammer A.A., Drake J., Heiner C., Clum A., Copeland A., Huddleston J., Eichler E.E. et al. Nonhybrid, finished microbial genome assemblies from long-read SMRT sequencing data. Nat. Methods. 2013; 10:563–569. 23644548 10.1038/nmeth.2474

[8] Vollger M.R., Logsdon G.A., Audano P.A., Sulovari A., Porubsky D., Peluso P., Wenger A.M., Concepcion G.T., Kronenberg Z.N., Munson K.M. et al. Improved assembly and variant detection of a haploid human genome using single-molecule, high-fidelity long reads. Ann. Hum. Genet. 2020; 84:125–140. 31711268 10.1111/ahg.12364 PMC7015760

[9] Logsdon G.A., Vollger M.R., Eichler E.E. Long-read human genome sequencing and its applications. Nat. Rev. Genet. 2020; 21:597–614. 32504078 10.1038/s41576-020-0236-x PMC7877196

[10] Sedlazeck F.J., Lee H., Darby C.A., Schatz M.C. Piercing the dark matter: bioinformatics of long-range sequencing and mapping. Nat. Rev. Genet. 2018; 19:329–346. 29599501 10.1038/s41576-018-0003-4

[11] Makałowski W., Shabardina V. Bioinformatics of nanopore sequencing. J. Hum. Genet. 2020; 65:61–67. 31451715 10.1038/s10038-019-0659-4

[12] Amarasinghe S.L., Su S., Dong X., Zappia L., Ritchie M.E., Gouil Q. Opportunities and challenges in long-read sequencing data analysis. Genome Biol. 2020; 21:30. 32033565 10.1186/s13059-020-1935-5 PMC7006217

[13] Escalona M., Rocha S., Posada D. A comparison of tools for the simulation of genomic next-generation sequencing data. Nat. Rev. Genet. 2016; 17:459–469. 27320129 10.1038/nrg.2016.57 PMC5224698

[14] Alosaimi S., Bandiang A., van Biljon N., Awany D., Thami P.K., Tchamga M.S. S., Kiran A., Messaoud O., Hassan R.I. M., Mugo J. et al. A broad survey of DNA sequence data simulation tools. Brief. Funct. Genomics. 2020; 19:49–59. 31867604 10.1093/bfgp/elz033 PMC7030445

[15] Ono Y., Asai K., Hamada M. PBSIM: PacBio reads simulator — toward accurate genome assembly. Bioinformatics. 2013; 29:119–121. 23129296 10.1093/bioinformatics/bts649

[16] Eid J., Fehr A., Gray J., Luong K., Lyle J., Otto G., Peluso P., Rank D., Baybayan P., Bettman B. et al. Real-time DNA sequencing from single polymerase molecules. Science. 2009; 323:133–138. 19023044 10.1126/science.1162986

[17] Ross M.G., Russ C., Costello M., Hollinger A., Lennon N.J., Hegarty R., Nusbaum C., Jaffe D.B. Characterizing and measuring bias in sequence data. Genome Biol. 2013; 14:R51. 23718773 10.1186/gb-2013-14-5-r51 PMC4053816

[18] Laehnemann D., Borkhardt A., McHardy A.C. Denoising DNA deep sequencing data—high-throughput sequencing errors and their correction. Brief. Bioinform. 2016; 17:154–179. 26026159 10.1093/bib/bbv029 PMC4719071

[19] Lau B., Mohiyuddin M., Mu J.C., Fang L.T., Bani Asadi N., Dallett C., Lam H.Y. LongISLND: in silico sequencing of lengthy and noisy datatypes. Bioinformatics. 2016; 32:3829–3832. 27667791 10.1093/bioinformatics/btw602 PMC5167071

[20] Zhang W., Jia B., Wei C. PaSS: A sequencing simulator for PacBio sequencing. BMC Bioinf. 2019; 20:352. 10.1186/s12859-019-2901-7 PMC6588853 31226925

[21] Wick R.R. Badread: simulation of error-prone long reads. J. Open Source Software. 2019; 4:1316.

[22] Faucon P.C., Balachandran P., Crook S. SNaResim: synthetic nanopore read simulator. 2017 IEEE International Conference on Healthcare Informatics (ICHI). 2017; Park City, UT, USA 338–344.

[23] Ono Y., Asai K., Hamada M. PBSIM2: a simulator for long-read sequencers with a novel generative model of quality scores. Bioinformatics. 2021; 37:589–595. 32976553 10.1093/bioinformatics/btaa835 PMC8097687

[24] Tvedte E.S., Gasser M., Sparklin B.C., Michalski J., Hjelmen C.E., Johnston J.S., Zhao X., Bromley R., Tallon L.J., Sadzewicz L. et al. Comparison of long read sequencing technologies in interrogating bacteria and fly genomes. G3 (Bethesda). 2021; 11:jkab083. 33768248 10.1093/g3journal/jkab083 PMC8495745

[25] Nurk S., Koren S., Rhie A., Rautiainen M., Bzikadze A.V., Mikheenko A., Vollger M.R., Altemose N., Uralsky L., Gershman A. et al. The complete sequence of a human genome. Science. 2022; 376:44–53. 35357919 10.1126/science.abj6987 PMC9186530

[26] Zook J.M., McDaniel J., Olson N.D., Wagner J., Parikh H., Heaton H., Irvine S.A., Trigg L., Truty R., McLean C.Y. et al. An open resource for accurately benchmarking small variant and reference calls. Nat. Biotechnol. 2019; 37:561–566. 30936564 10.1038/s41587-019-0074-6 PMC6500473

[27] Koren S., Schatz M.C., Walenz B.P., Martin J., Howard J.T., Ganapathy G., Wang Z., Rasko D.A., McCombie W.R., Jarvis E.D. et al. Reducing assembly complexity of microbial genomes with single-molecule sequencing. Genome Biol. 2013; 14:R101. 24034426 10.1186/gb-2013-14-9-r101 PMC4053942

[28] Chen Y., Davidson N., Wan Y.K., Patel H., Yao F., Low H.M., Hendra C., Watten L., Sim A., Sawyer C. et al. A systematic benchmark of Nanopore long read RNA sequencing for transcript level analysis in human cell lines. 2021; bioRxiv doi:22 April 2021, preprint: not peer reviewed 10.1101/2021.04.21.440736. PMC11978509 40082608

[29] Mitsuhashi S., Nakagawa S., Sasaki-Honda M., Sakurai H., Frith M.C., Mitsuhashi H. Nanopore direct RNA sequencing detects DUX4-activated repeats and isoforms in human muscle cells. Hum. Mol. Genet. 2021; 30:552–563. 33693705 10.1093/hmg/ddab063 PMC8120133

[30] Kiełbasa S.M., Wan R., Sato K., Horton P., Frith M.C. Adaptive seeds tame genomic sequence comparison. Genome Res. 2011; 21:487–493. 21209072 10.1101/gr.113985.110 PMC3044862

[31] Frith M.C., Kawaguchi R. Split-alignment of genomes finds orthologies more accurately. Genome Biol. 2015; 16:106. 25994148 10.1186/s13059-015-0670-9 PMC4464727

[32] Hamada M., Ono Y., Asai K., Frith M.C. Training alignment parameters for arbitrary sequencers with LAST-TRAIN. Bioinformatics. 2017; 33:926–928. 28039163 10.1093/bioinformatics/btw742 PMC5351549

[33] Yates A.D., Achuthan P., Akanni W., Allen J., Alvarez-Jarreta J., Amode M.R., Armean I.M., Azov A.G., Bennett R., Bhai J. et al. Ensembl 2020. Nucleic Acids Res. 2020; 48:D682–D688. 31691826 10.1093/nar/gkz966 PMC7145704

[34] Li H. Minimap2: pairwise alignment for nucleotide sequences. Bioinformatics. 2018; 34:3094–3100. 29750242 10.1093/bioinformatics/bty191 PMC6137996

[35] Hamada M., Ono Y., Fujimaki R., Asai K. Learning chromatin states with factorized information criteria. Bioinformatics. 2015; 31:2426–2433. 25810430 10.1093/bioinformatics/btv163

[36] Fujimaki R., Hayashi K. Factorized asymptotic Bayesian hidden Markov models. 2012; arXiv doi:18 June 2012, preprint: not peer reviewed https://arxiv.org/abs/1206.4679.

[37] Yang C., Chu J., Warren R.L., Birol I. NanoSim: nanopore sequence read simulator based on statistical characterization. GigaScience. 2017; 6:gix010. 10.1093/gigascience/gix010 PMC5530317 28327957

[38] Seki M., Katsumata E., Suzuki A., Sereewattanawoot S., Sakamoto Y., Mizushima-Sugano J., Sugano S., Kohno T., Frith M.C., Tsuchihara K. et al. Evaluation and application of RNA-Seq by MinION. DNA Res. 2019; 26:55–65. 30462165 10.1093/dnares/dsy038 PMC6379022

[39] Delahaye C., Nicolas J. Sequencing DNA with nanopores: troubles and biases. PLoS One. 2021; 16:e0257521. 34597327 10.1371/journal.pone.0257521 PMC8486125

[40] Wick R.R., Judd L.M., Holt K.E. Performance of neural network basecalling tools for Oxford Nanopore sequencing. Genome Biol. 2019; 20:129. 31234903 10.1186/s13059-019-1727-y PMC6591954

[41] Stöcker B.K., Köster J., Rahmann S. SimLoRD: simulation of long read data. Bioinformatics. 2016; 32:2704–2706. 27166244 10.1093/bioinformatics/btw286

[42] Dierckxsens N., Li T., Vermeesch J.R., Xie Z. A benchmark of structural variation detection by long reads through a realistic simulated model. Genome Biol. 2021; 22:342. 34911553 10.1186/s13059-021-02551-4 PMC8672642

[43] Weirather J.L., de Cesare M., Wang Y., Piazza P., Sebastiano V., Wang X.J., Buck D., Au K.F. Comprehensive comparison of Pacific Biosciences and Oxford Nanopore Technologies and their applications to transcriptome analysis. F1000Res. 2017; 6:100. 28868132 10.12688/f1000research.10571.1 PMC5553090

[44] Namba S., Ueno T., Kojima S., Kobayashi K., Kawase K., Tanaka Y., Inoue S., Kishigami F., Kawashima S., Maeda N. et al. Transcript-targeted analysis reveals isoform alterations and double-hop fusions in breast cancer. Commun. Biol. 2021; 4:1320. 34811492 10.1038/s42003-021-02833-4 PMC8608905

[45] Hafezqorani S., Yang C., Lo T., Nip K.M., Warren R.L., Birol I. Trans-NanoSim characterizes and simulates nanopore RNA-sequencing data. GigaScience. 2020; 9:giaa061. 32520350 10.1093/gigascience/giaa061 PMC7285873

[46] Stark R., Grzelak M., Hadfield J. RNA sequencing: the teenage years. Nat. Rev. Genet. 2019; 20:631–656. 31341269 10.1038/s41576-019-0150-2

[47] Kuo R.I., Cheng Y., Zhang R., Brown J.W., Smith J., Archibald A.L., Burt D.W. Illuminating the dark side of the human transcriptome with long read transcript sequencing. BMC genomics. 2020; 21:751. 33126848 10.1186/s12864-020-07123-7 PMC7596999

[48] Hu Y., Fang L., Chen X., Zhong J.F., Li M., Wang K. LIQA: long-read isoform quantification and analysis. Genome Biol. 2021; 22:182. 34140043 10.1186/s13059-021-02399-8 PMC8212471

[49] Gleeson J., Leger A., Prawer Y.D., Lane T.A., Harrison P.J., Haerty W., Clark M.B. Accurate expression quantification from nanopore direct RNA sequencing with NanoCount. Nucleic Acids Res. 2022; 50:e19. 34850115 10.1093/nar/gkab1129 PMC8886870

[50] Hoyt S.J., Storer J.M., Hartley G.A., Grady P.G. S., Gershman A., de Lima L.G., Limouse C., Halabian R., Wojenski L., Rodriguez M. et al. From telomere to telomere: The transcriptional and epigenetic state of human repeat elements. Science. 2022; 376:eabk3112. 35357925 10.1126/science.abk3112 PMC9301658

[51] Shi H., Zhou Y., Jia E., Pan M., Bai Y., Ge Q. Bias in RNA-seq library preparation: current challenges and solutions. Biomed Res. Int. 2021; 2021:6647597. 33987443 10.1155/2021/6647597 PMC8079181

