# Supplementary material for: PBSIM3: a simulator for all types of PacBio and ONT long reads
Source: NAR Genom Bioinform. 2022 Dec 1;4(4):lqac092. doi: 10.1093/nargab/lqac092 (PMC9713900; doi:10.1093/nargab/lqac092)
Supplement: lqac092_Supplemental_File [file lqac092_supplemental_file.pdf]

Supplementary Information of
"PBSIM3: a simulator for all types of PacBio and ONT long reads"

Yukiteru Ono *, Michiaki Hamada and Kiyoshi Asai*

A Supplementary Tables

*To whom correspondence should be addressed. Tel: +814 7136 3986; Fax: +814 7136 4074; Email: yono@k.u-tokyo.ac.jp
Correspondence may also be addressed to Kiyoshi Asai. Tel: +814 7136 3986; Fax: +814 7136 4074; Email: asai@k.u-tokyo.ac.jp

Table S1: Datasets for whole genome sequencing

Sequencer + Type	Species	Read length	Read accuracy	URL
PacBio RS II CLR	*Caenorhabditis elegans*	11,560	85.91%	https://github.com/PacificBiosciences/DevNet/wiki/C.-elegans-data-set
	Escherichia coli K12	8,562	85.16%	https://github.com/PacificBiosciences/DevNet/wiki/E.-coli-Bacterial-Assembly
PacBio Sequel CLR	*E. coli* K12	11,482	–	https://github.com/PacificBiosciences/DevNet/wiki/Datasets
	Homo sapiens	23,517	–	https://trace.ncbi.nlm.nih.gov/Traces/sra/?run=ERR5101156 [a]
	H. sapiens CHM13	17,769	–	https://s3-us-west-2.amazonaws.com/human-pangenomics/T2T/CHM13/pacbio/hifi_20kb/m64062_190803_042216.subreads.bam [a]
ONT R9.4	*H. sapiens* CHM13	24,358	77.02%	https://s3-us-west-2.amazonaws.com/human-pangenomics/T2T/CHM13/nanopore/rel8-guppy-5.0.7/reads.fastq.gz
	Drosophila melanogaster	16,325	92.47%	https://trace.ncbi.nlm.nih.gov/Traces/sra/?run=SRR13070625
ONT R10.3	*E. coli* K12	6,397	85.09%	https://trace.ncbi.nlm.nih.gov/Traces/sra/?run=ERR3890216
	E. coli O127	8,513	82.80%	https://trace.ncbi.nlm.nih.gov/Traces/sra/?run=SRR13610060
PacBio Sequel HiFi	*H. sapiens* CHM13	20,716	99.81%	https://trace.ncbi.nlm.nih.gov/Traces/sra/?run=SRR11292120 [b]
	E. coli K12	14,548	99.78%	https://trace.ncbi.nlm.nih.gov/Traces/sra/?run=SRR10971019

Read accuracy was computed from quality scores.
[a] 100,000 reads were sampled.
[b] 10,000 reads with a length of 1,000 bp or more were sampled

Table S2: Datasets for transcriptome sequencing

Sequencer + Type	Species	Read length	Read accuracy	URL
PacBio Iso-seq	*H. sapiens* Alzheimer's disease	2,919	–	https://www.pacb.com/smrt-science/smrt-resources/datasets/
	H. sapiens UHRR	3,185	99.90%	https://www.pacb.com/smrt-science/smrt-resources/datasets/
ONT direct RNA	*H. sapiens* GM12878 basecalled by Guppy 4.2.2	962	87.95%	https://github.com/nanopore-wgs-consortium/NA12878
	H. sapiens A549	976	81.59%	https://www.ebi.ac.uk/ena/browser/view/PRJEB44348 [a]
	H. sapiens RD cell	1,059	80.32%	https://trace.ncbi.nlm.nih.gov/Traces/sra/?run=DRR178487
ONT direct cDNA	*H. sapiens* GM12878 basecalled by Guppy 4.2.2	944	88.47%	https://github.com/nanopore-wgs-consortium/NA12878
	H. sapiens A549	876	88.71%	https://trace.ncbi.nlm.nih.gov/Traces/sra/?run=ERR6053016

Read accuracy was computed from quality scores.
1,000,000 reads with a length of 50 bp or more were sampled.
[a] The number of sampled reads is 534,521.

Table S3: Alignment statistics of whole genome sequencing

Sequencer + Type	Species	Aligned rate(read)	Aligned rate(base)	Sub. rate	Ins. rate	Del. rate	Total	Reference
PacBio RS II CLR	*C. elegans*	98.27%	95.51%	0.63%	5.71%	4.06%	10.39%	Assembly ce10 (version WBcel215)
	E. coli K12	97.77%	92.87%	0.67%	7.96%	3.62%	12.25%	GenBank-LOCUS:NC_000913
PacBio Sequel CLR	*E. coli* K12	99.83%	97.11%	2.18%	4.56%	3.37%	10.11%	GenBank-LOCUS:NC_000913
	H. sapiens	96.61%	89.82%	3.73%	4.70%	3.95%	12.38%	Genome Reference Consortium Human GRCh38.p13
	H. sapiens CHM13	99.17%	97.87%	3.03%	4.06%	3.54%	10.64%	Complete T2T reconstruction of a human genome v1.1 (25)
ONT R9.4	*H. sapiens* CHM13	95.81%	70.05%	5.89%	5.62%	9.03%	20.54%	Complete T2T reconstruction of a human genome v1.1 (25)
	D. melanogaster	99.97%	97.79%	1.65%	2.82%	4.94%	9.41%	Assembly dm6
ONT R10.3	*E. coli* K12	99.92%	98.59%	5.58%	3.63%	4.87%	14.08%	GenBank-LOCUS:NC_000913
	E. coli O127	96.89%	95.63%	5.76%	3.56%	5.27%	14.59%	GenBank-LOCUS:NC_011601
PacBio Sequel HiFi	*H. sapiens* CHM13	100.00%	99.98%	0.01%	0.10%	0.13%	0.25%	Complete T2T reconstruction of a human genome v1.1 (25)
	E. coli K12	100.00%	99.99%	0.01%	0.11%	0.11%	0.22%	GenBank-LOCUS:NC_000913

Sub.: Substitution, Ins.: Insertion, Del.: Deletion.
Statistics were calculated from the alignments between the reads and their reference genomes.

Table S4: Parameter settings of aligners and simulators

Aligner/Simulator	Function	Parameter settings
LAST	read alignment	`lastal -k8 -c2 -l30` for HiFi reads.
texttt lastal use the default settings for CLR and ONT reads.		
		`lastdb, last-train, and last-split` use the default settings.
Minimap2	read alignment	`minimap2 --cs=long -k 19 -O 5,56 -E 4,1 -B 5 -z 400,50 -r 2k --eqx --secondary=no`
PBSIM3	WGS simulation	(when using quality score model) `pbsim --strategy wgs --method qshmm --qshmm` *`quality-score-model`* `--length-mean 15000 --length-sd 15000 --accuracy-mean 0.85 --difference-ratio` *`ratio`*
	TS simulation	(when using error model) `pbsim --strategy trans --method errhmm --errhmm` *`error-model`* `--length-mean 15000 --length-sd 15000 --accuracy-mean 0.85`
Badread	WGS simulation	(to simulate PacBio reads) `badread simulate --length 15000,15000 --identity 85,97.5,7 --error model pacbio2016 --qscore model pacbio2016` (to simulate ONT reads) `badread simulate --length 15000,15000 --identity 85,97.5,7 --error model nanopore2020 --qscore model nanopore2020`
NanoSim	WGS simulation	`simulator.py genome -b guppy -k 6 -s 0.5 -c human_NA12878_DNA_FAB49712_guppy/tra`

Table S5: Alignment statistics of transcriptome sequencing

Sequencer + Type	Species	Aligned rate(read)	Aligned rate(base)	Sub. rate	Ins. rate	Del. rate	Total
PacBio Sequel Iso-seq	H. sapiens Alzheimer's disease	95.64%	71.22%	0.64%	0.48%	1.41%	2.53%
	H. sapiens UHRR	99.11%	80.23%	0.26%	0.11%	0.13%	0.49%
ONT direct RNA	H. sapiens GM12878 basecalled by Guppy 4.2.2	85.02%	86.17%	2.17%	2.60%	5.99%	10.76%
	H.sapiens A549	82.91%	79.19%	3.98%	4.56%	8.61%	17.15%
	H. sapiens RD cell	71.76%	72.26%	5.01%	3.85%	9.61%	18.47%
ONT direct cDNA	H. sapiens GM12878	89.56%	75.80%	2.34%	2.66%	5.34%	10.34%
	H. sapiens A549	98.54%	85.04%	2.79%	4.40%	6.19%	13.38%

Sub.: Substitution, Ins.: Insertion, Del.: Deletion.
Statistics were calculated from the alignments between the reads and their reference genomes.

Table S6: Comparison of whole genome sequencing alignment statistics between real and simulated reads

Sequencer + Type	Species	Real or Simulator	Sub. rate	Ins. rate	Del. rate	Total
PacBio RS II CLR	*C. elegans*	Real reads	0.63%	5.71%	4.06%	10.39%
		Random model	1.68%	6.10%	3.83%	11.61%
		Quality score model	1.68%	5.96%	3.68%	11.32%
		Error model	0.86%	7.75%	5.36%	13.97%
PacBio Sequel CLR	*E. coli* K12	Real reads	2.18%	4.56%	3.37%	10.11%
		Error model	3.17%	6.29%	4.24%	13.70%
		Badread	2.86%	4.67%	6.53%	14.06%
ONT	*E. coli* O127	Real reads	5.76%	3.56%	5.27%	14.59%
		Quality score model	5.70%	2.28%	3.99%	11.97%
		Error model	5.78%	3.24%	5.00%	14.02%
		Badread	4.82%	2.89%	6.98%	14.69%
		NanoSim	2.41%	2.46%	5.33%	10.20%

Sub.: Substitution, Ins.: Insertion, Del.: Deletion.
The error rates were calculated from the alignments between the reads and their reference genomes.

Table S7: Simulation of HiFi reads (*E. coli* K12)

Real or simulators	CLR error rate	Sub. rate	Ins. rate	Del. rate	Total
Real reads		0.01%	0.11%	0.11%	0.22%
Random model	10%	0.00%	0.06%	0.07%	0.13%
	15%	0.01%	0.15%	0.16%	0.31%
	20%	0.03%	0.28%	0.32%	0.63%
Quality score model (RS II)	10%	0.00%	0.04%	0.06%	0.10%
	15%	0.01%	0.10%	0.13%	0.24%
	20%	0.02%	0.18%	0.27%	0.47%
Error model (RS II)	10%	0.00%	0.09%	0.10%	0.19%
	15%	0.00%	0.19%	0.27%	0.47%
	20%	0.01%	0.39%	0.60%	1.01%
Error model (Sequel)	10%	0.00%	0.04%	0.12%	0.17%
	15%	0.01%	0.10%	0.32%	0.43%
	20%	0.03%	0.18%	0.80%	1.01%

Sub.: Substitution, Ins.: Insertion, Del.: Deletion.
The reference genome is *E. coli* K12. Simulated HiFi reads were generated by ccs software as consensus sequences from simulated CLR reads. The error rates were calculated from the alignments between the reads and their reference genomes.

Table S8: The effect of the number of passes on the simulation of PacBio HiFi reads

Real or simulators	Number of passes	CCS yield	Sub. rate	Ins. rate	Del. rate	Total
(A) *H. sapiens* CHM13						
Real reads			0.02%	0.10%	0.13%	0.25%
Quality score model (RS II)	5	23.73%	0.09%	0.46%	0.44%	0.99%
	10	98.27%	0.01%	0.12%	0.20%	0.34%
	15	99.20%	0.01%	0.05%	0.12%	0.17%
	20	99.52%	0.00%	0.03%	0.08%	0.12%
(B) *E. coli* K12						
Real reads			0.01%	0.11%	0.11%	0.22%
Quality score model (RS II)	5	37.29%	0.08%	0.46%	0.37%	0.91%
	10	98.60%	0.01%	0.10%	0.13%	0.24%
	15	99.40%	0.00%	0.03%	0.06%	0.09%
	20	99.13%	0.00%	0.01%	0.04%	0.05%

Sub.: Substitution, Ins.: Insertion, Del.: Deletion.
CCS yield: The rate at which ccs software was able to generate HiFi reads from CLR reads.
Simulated HiFi reads were generated by ccs software as consensus sequences from simulated CLR reads. The error rate of CLR was 15%. We tried four number of passes: 5, 10, 15, and 20. The error ratio is 22:45:33. The length of all CLR reads was 15 kb. The error rates were calculated from the alignments between the reads and their reference genomes.

B Supplementary Figures

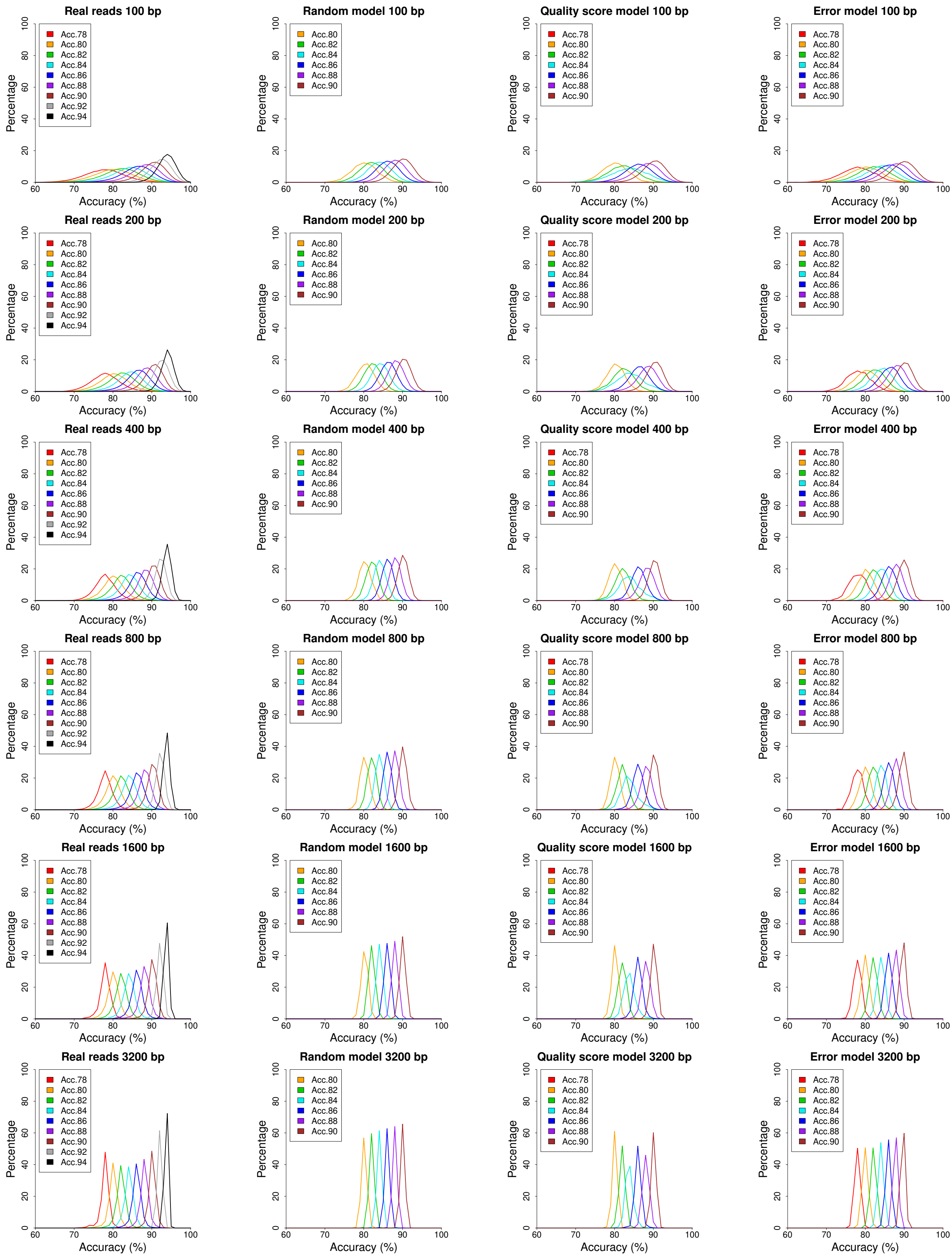

Figure S1: Non-uniformity of errors of PacBio RS II CLR reads for *C. elegans*. After grouping reads by their accuracy, they were segmented into fixed size (100, 200, 400, 800, 1600, and 3200 bp) disjoint intervals, and accuracy of each interval was computed. Each graph shows the distribution of the averaged accuracy of each intervals, where the color of the plotted lines represents read groups (e.g., 'Acc.78' refers to a read group with an accuracy of 77.5–78.4%). The random model randomly generates errors according to an error rate and error ratio.

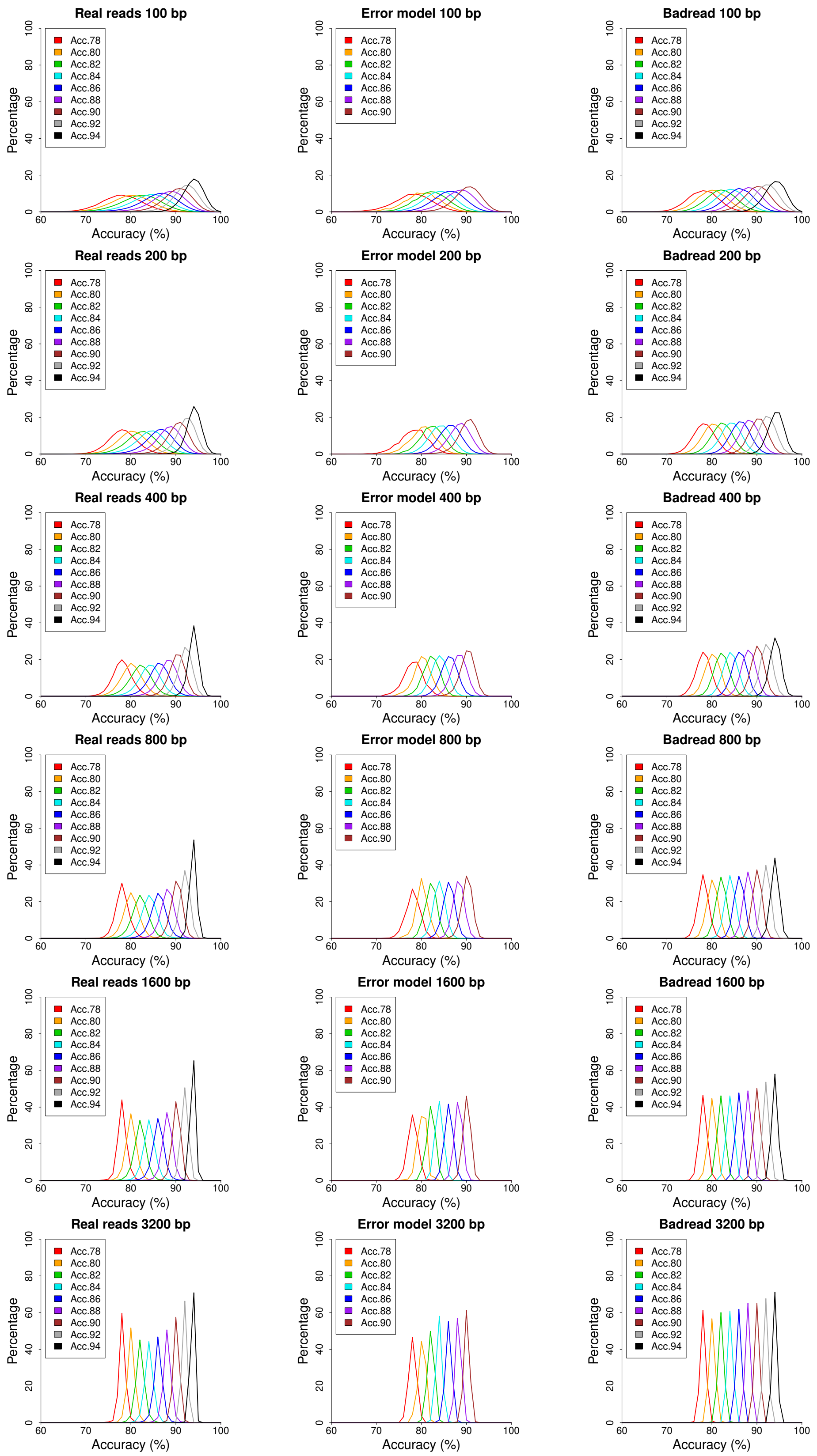

Figure S2: Non-uniformity of errors of PacBio Sequel CLR reads for *E. coli* K12. After grouping reads by their accuracy, they were segmented into fixed size (100, 200, 400, 800, 1600, and 3200 bp) disjoint intervals, and accuracy of each interval was computed. Each graph shows the distribution of the averaged accuracy of each intervals, where color of the plotted lines represents read groups (e.g., 'Acc.78' refers to a read group with an accuracy of 77.5–78.4%).

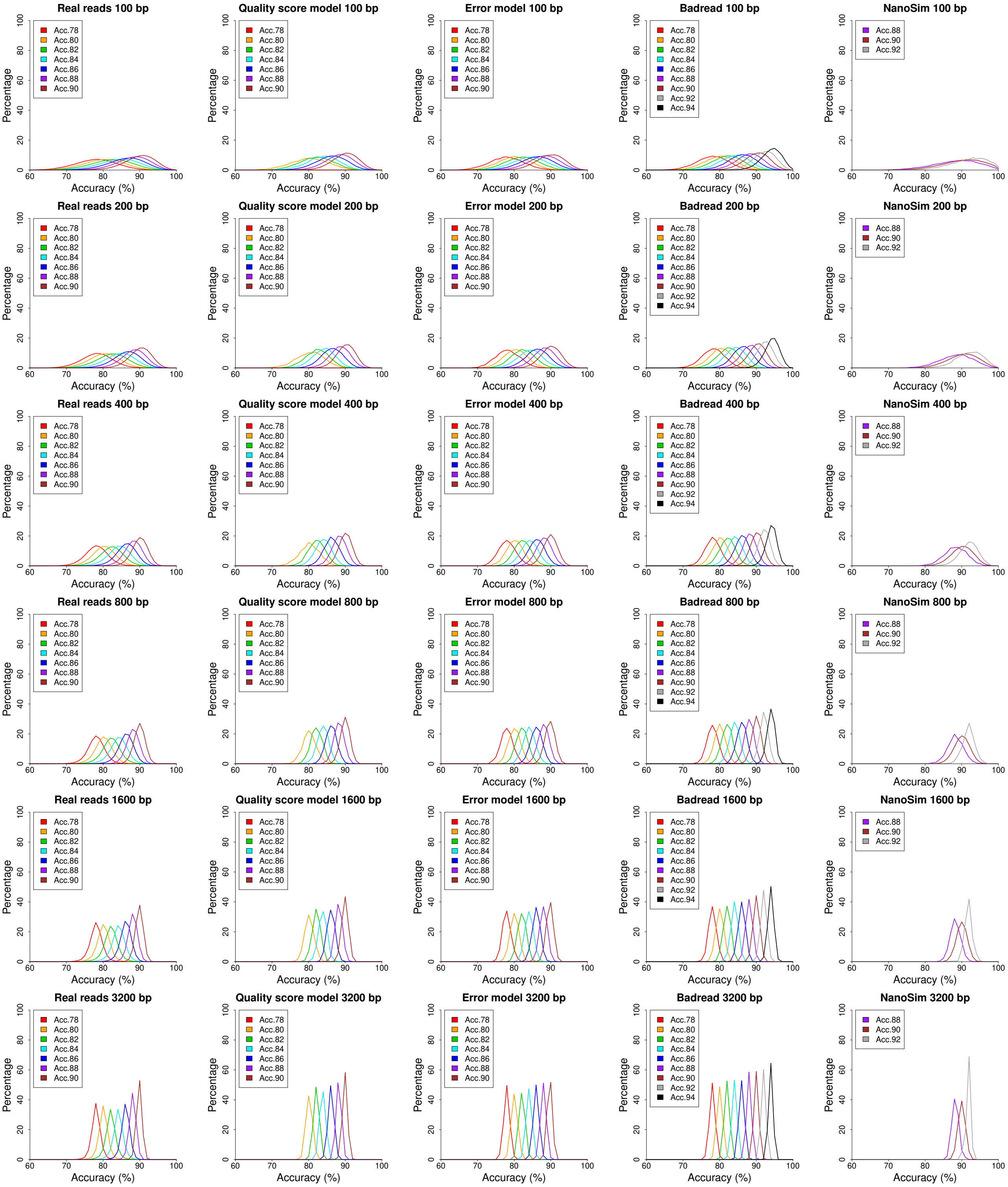

Figure S3: Non-uniformity of errors of ONT reads for *E. coli* O127. After grouping reads by their accuracy, they were segmented into fixed size (100, 200, 400, 800, 1600, and 3200 bp) disjoint intervals, and accuracy of each interval was computed. Each graph shows the distribution of the averaged accuracy of each intervals, where color of the plotted lines represents read groups (e.g., 'Acc.78' refers to a read group with an accuracy of 77.5–78.4%).

(A) PacBio RS II CLR reads for *C. elegans*

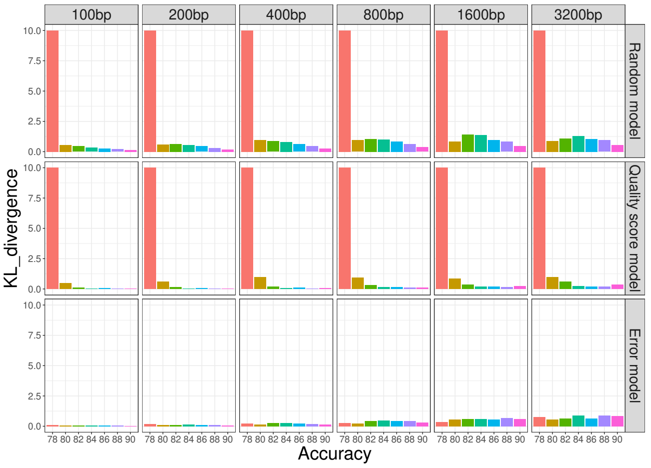

(B) PacBio Sequel CLR reads for *E. coli* K12

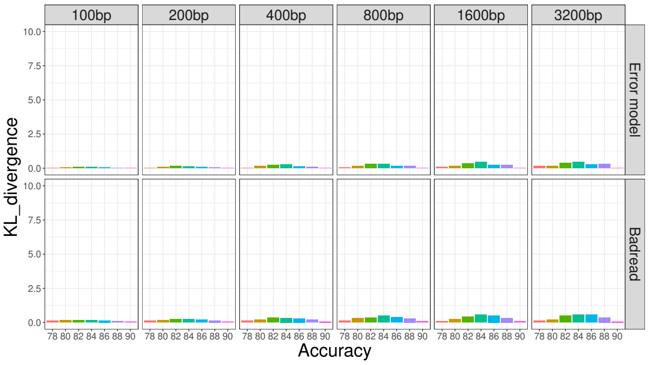

(C) ONT reads for *E. coli* O127

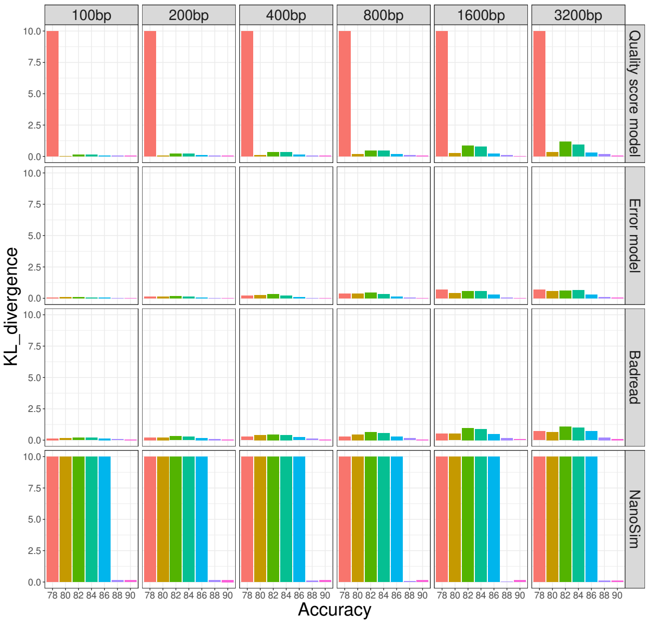

Figure S4: Evaluation of Non-uniformity of errors by Kullback-Leibler (KL) divergence. KL divergence of distribution of accuracy of fixed size (50, 100, 200, 400, 800, 1600 and 3200bp) intervals between real and simulated reads. Upper-limit value of KL divergence was 10.

(A) PacBio RS II CLR for *C. elegans*

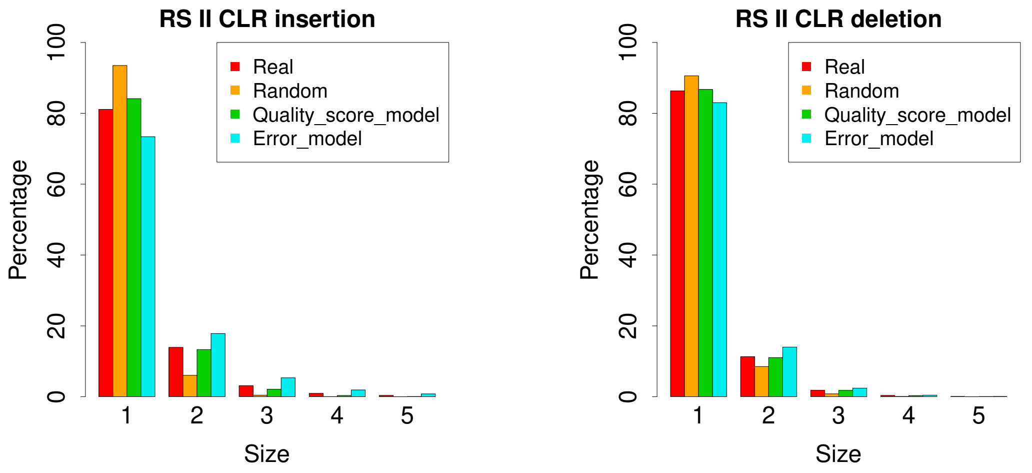

(B) PacBio Sequel CLR for *E. coli* K12

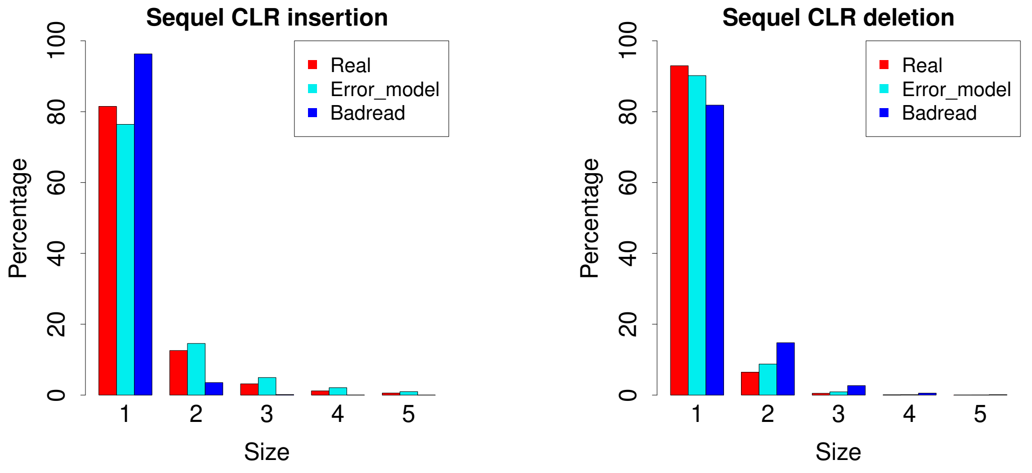

(C) ONT for *E. coli* O127

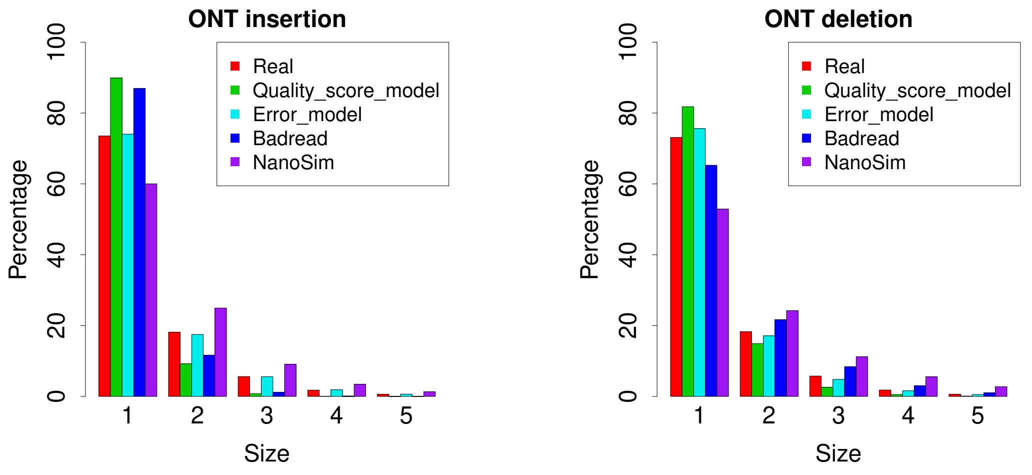

Figure S5: Distributions of insertion and deletion (indel) length for real reads and simulated reads. The vertical axis represents the percentage, while the horizontal axis represents the indel length. Frequencies of indel length were obtained from alignments between the reads and their reference genomes.

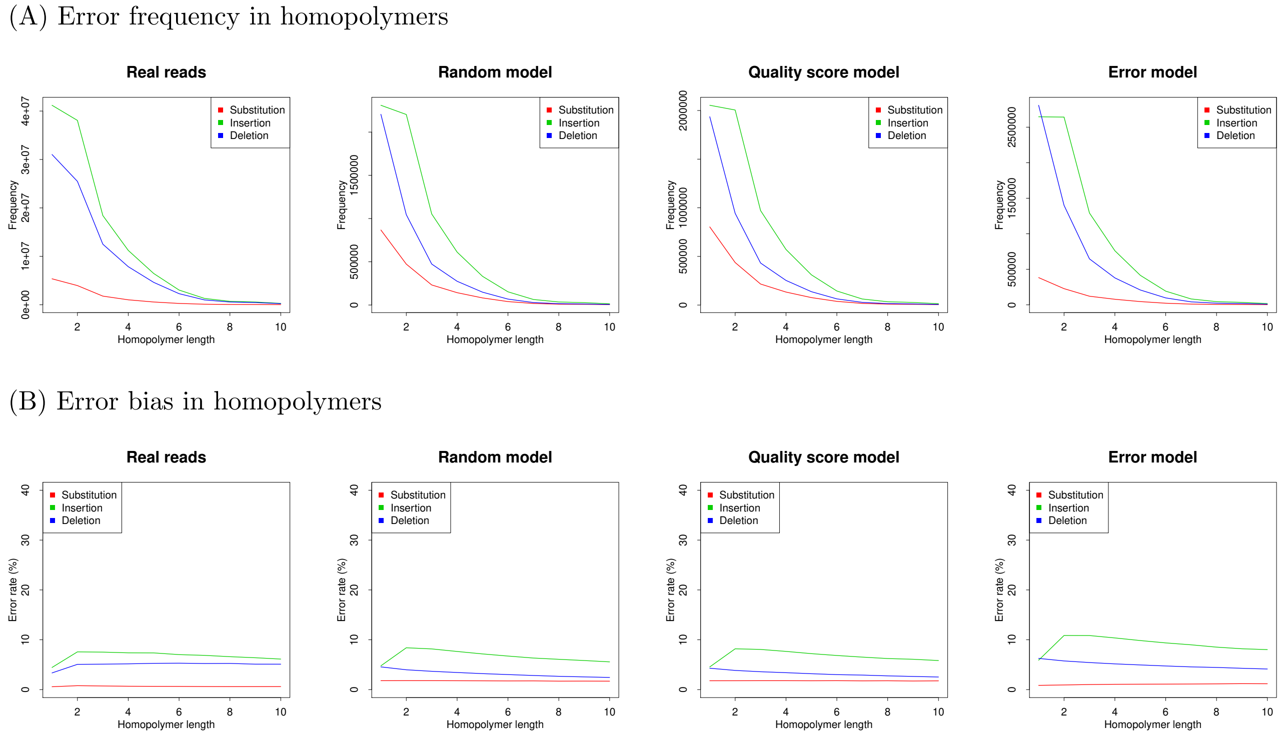

Figure S6: The error frequencies and bias in homopolymers of PacBio RS II CLR reads for *C. elegans*. If a site is contained in a genomic region where N identical bases are continuous, homopolymer length is designated as N. Then, for each of the homopolymer lengths, the number of errors was counted and error rate was calculated. CLR reads were simulated using the random, the quality score, and the error model. The error rates were calculated from alignments between the reads and their reference genomes.

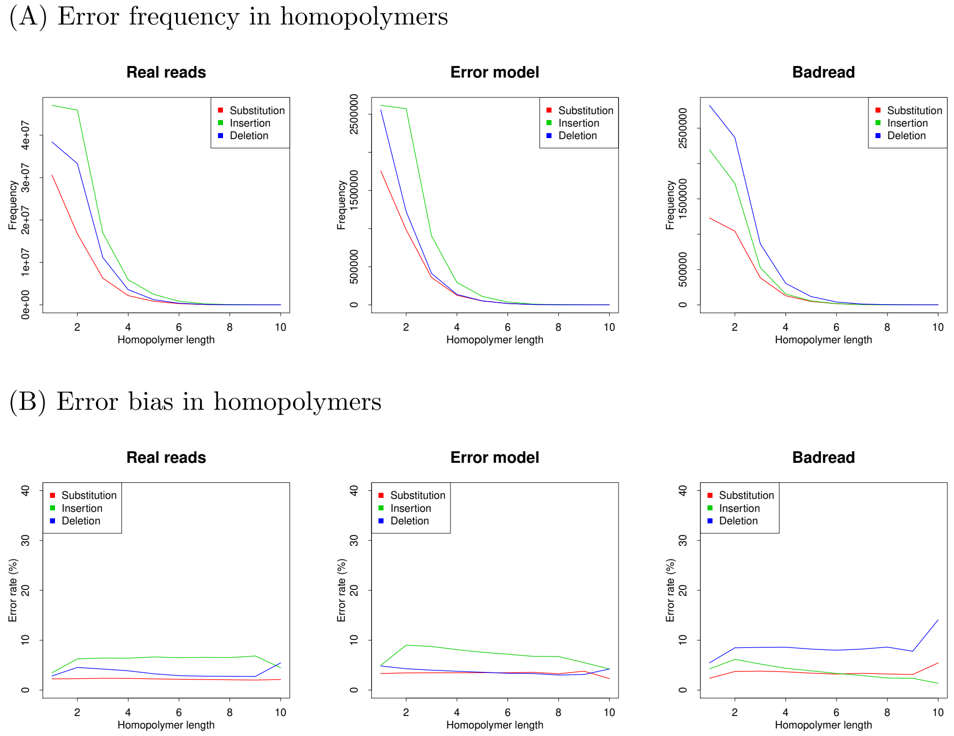

Figure S7: The error frequencies and bias in homopolymers of PacBio Sequel CLR reads for *E. coli* K12. If a site is contained in a genomic region where N identical bases are continuous, homopolymer length is designated as N. Then, for each of the homopolymer lengths, the number of errors was counted and error rate was calculated. CLR reads were simulated using the quality score model and Badread. The error rates were calculated from alignments between the reads and their reference genomes.

(A) Error frequency in homopolymers

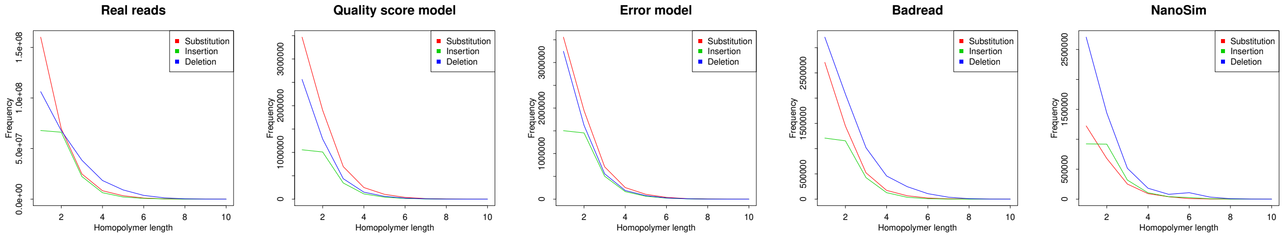

(B) Error bias in homopolymers

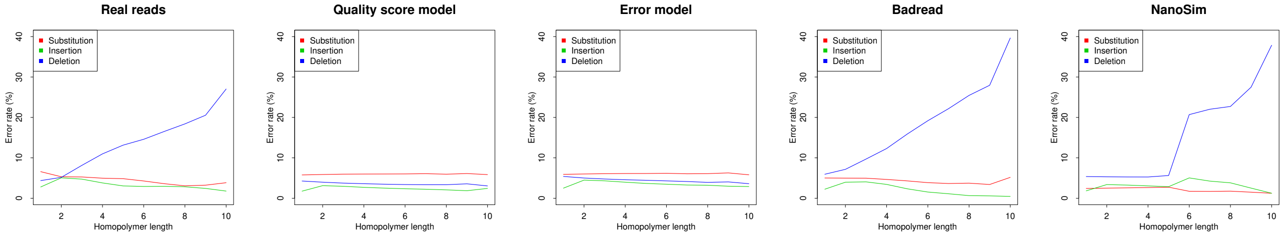

(C) Error bias in homopolymers (deletion homopolymer bias introduced)

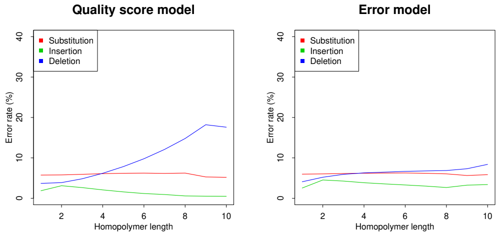

Figure S8: The error frequencies and bias in homopolymers of ONT reads for *E. coli* O127. If a site is contained in a genomic region where N identical bases are continuous, homopolymer length is designated as N. Then, for each of the homopolymer lengths, the number of errors was counted and error rate was calculated. ONT reads were simulated using the quality score model, the error model, Badread, and NanoSim. The deletion homopolymer bias was introduced with option '`--hp-del-bias 6`'. The error rates were calculated from alignments between the reads and their reference genomes.

(A) Error frequency in homopolymers for *E. coli* K12

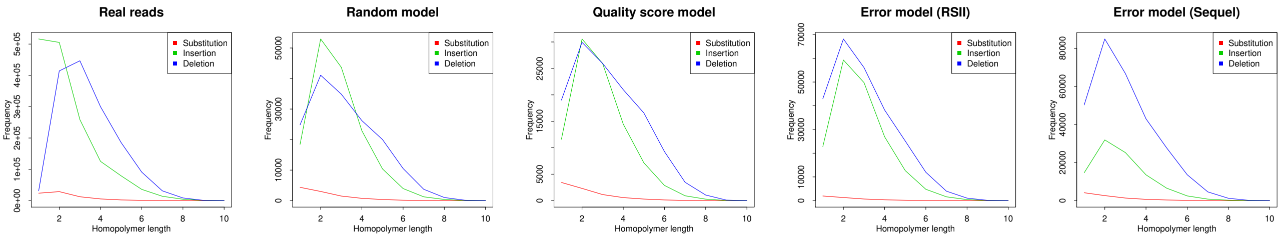

(B) Error bias in homopolymers for *E. coli* K12

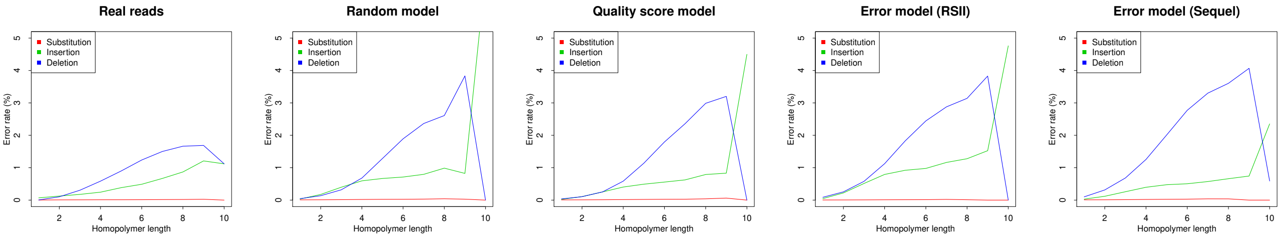

(C) Error frequency in homopolymers for *H. sapiens* CHM13

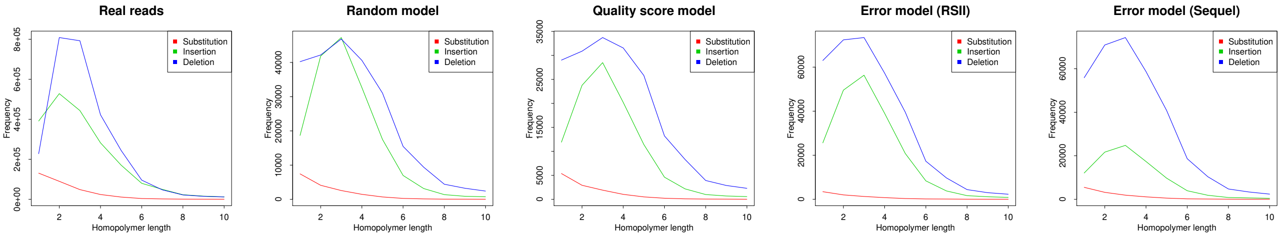

(D) Error bias in homopolymers for *H. sapiens* CHM13

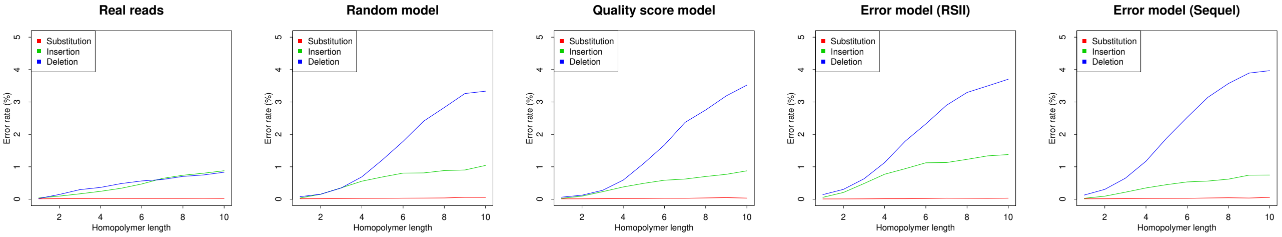

Figure S9: The error frequencies and bias in homopolymers of PacBio HiFi reads. If a site is contained in a genomic region where N identical bases are continuous, homopolymer length is designated as N. Then, for each of the homopolymer lengths, the number of errors was counted. The simulated reads were generated by ccs software as consensus sequences from CLR reads simulated using the PBSIM3 quality score model. The error rates were calculated from alignments between the reads and their reference genomes.

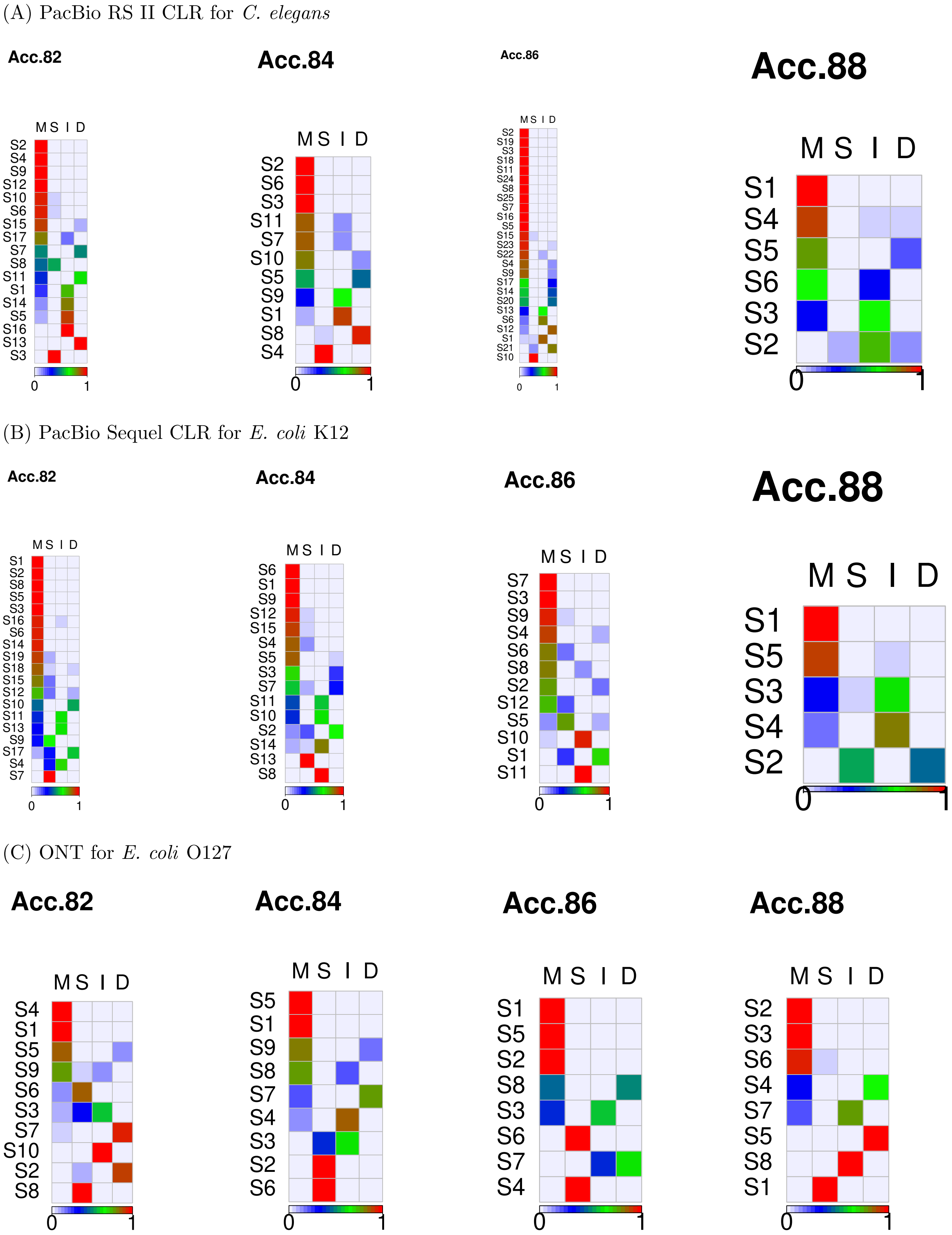

Figure S10: Emission probability matrices of states of FIC-HMM. The horizontal axis represents alignment states; M:Match, S:Substitution, I:Insertion, D:Deletion. The vertical axis represents states of FIC-HMM, which are sorted in descending order of M(atch) probability emitted by the states of FIC-HMM. The states on the vertical axis emit the alignment states on the horizontal axis. The sum of emission probabilities on each state of vertical axis is 100%. These are matrices of 'Acc.82'–'Acc.88' (e.g., 'Acc.84' refers to a read group with an accuracy of 83.5%–84.4%).

(A) PacBio RS II CLR for *C. elegans*

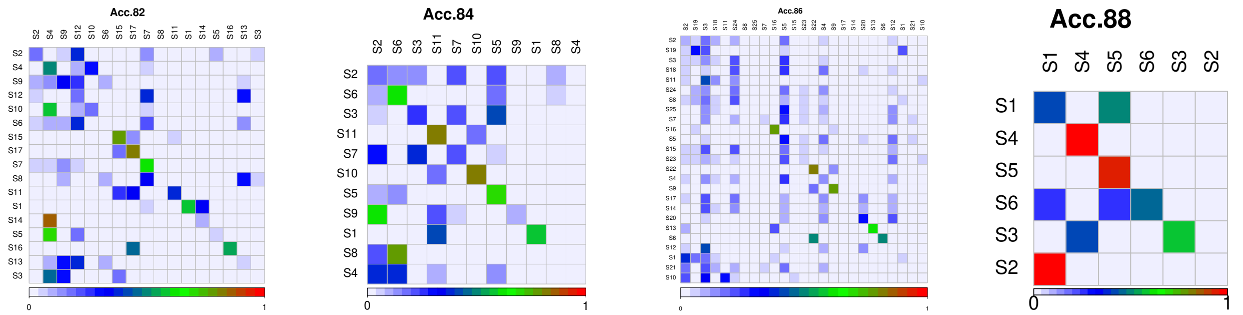

(B) PacBio Sequel CLR for *E. coli* K12

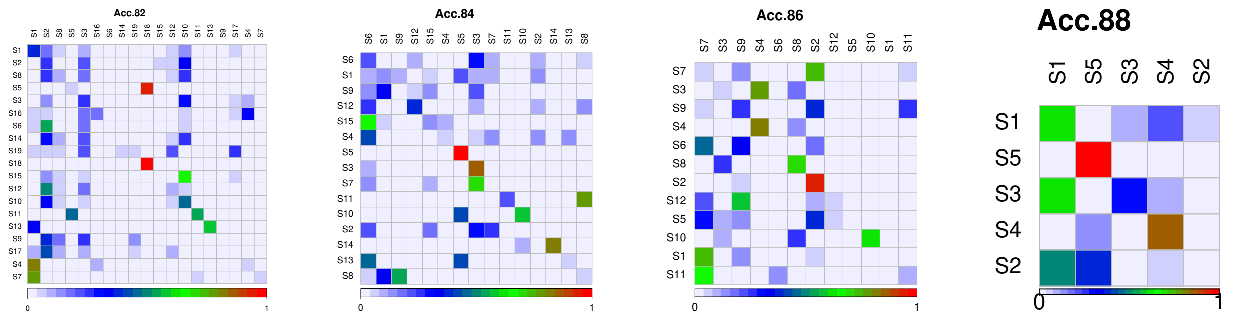

(C) ONT for *E. coli* O127

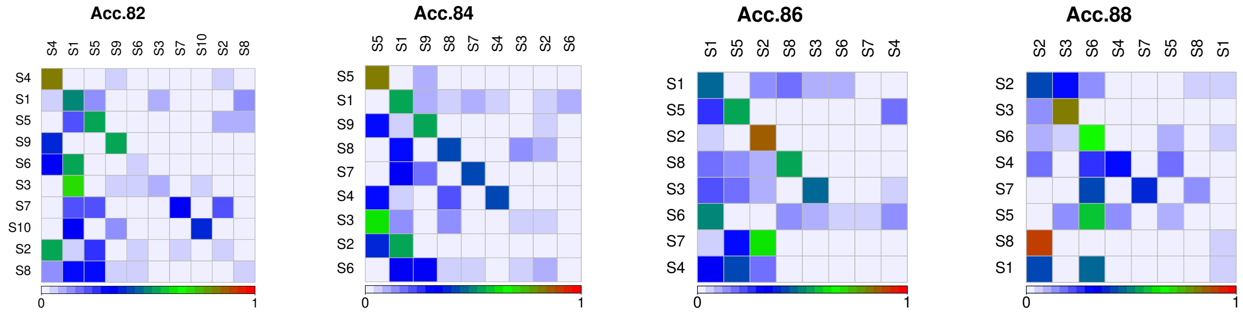

Figure S11: Transition probability matrices of states of FIC-HMM. The vertical and horizontal axes represent states of FIC-HMM, which are sorted in the same order as the emission probability matrices (Supplementary Figure S10). The states on the vertical axis transition to the states on the horizontal axis. The sum of transition probabilities on each state of the vertical axis is 100%. These are matrices of 'Acc.82'–'Acc.88' (e.g., 'Acc.84' refers to a read group with an accuracy of 83.5%–84.4%).

(A) Real reads

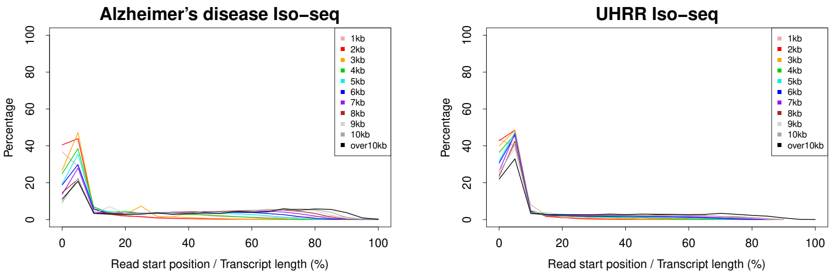

(B) Simulated reads

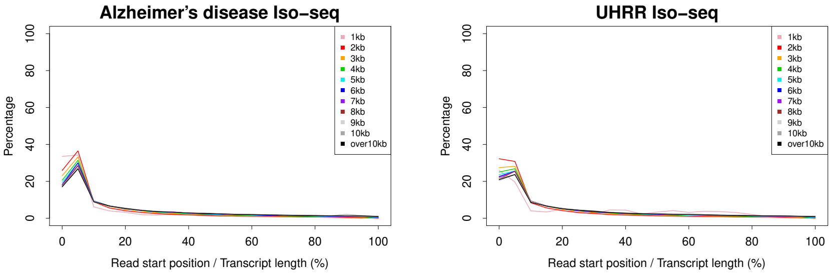

Figure S12: Read start positions of PacBio Iso-seq (HiFi reads) on their template transcripts. Reads were grouped by 1 kp by their template length. Each graph shows the distribution of the read start positions, where colors of plotted lines represent read groups (e.g., '1kb' refers to a read group with their template length of 1–1000 bp). The horizontal axis indicates the position of the read start positions in the total length of their templates, which was calculated by dividing the read start position by the total length of the template; the graph is plotted in 5% increments, with the left edge of the graph showing the percentage of reads starting exactly at the 5'end of the template. CLR reads were simulated using the PBSIM3 quality score models, and HiFi reads were generated by ccs software as consensus sequences from PBSIM3 outputs. The template transcript from which each read was most likely sequenced and the read start position was obtained from alignments between the reads and their reference transcriptomes.

(A) Real reads

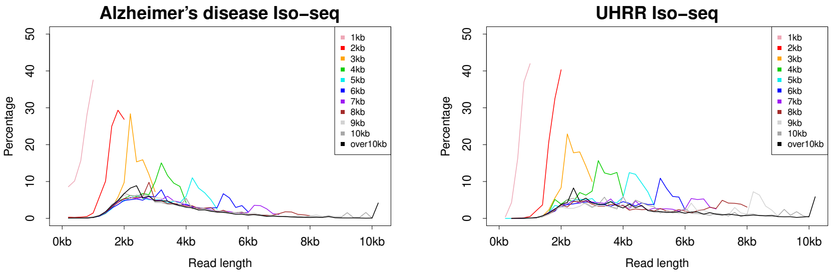

(B) Simulated reads

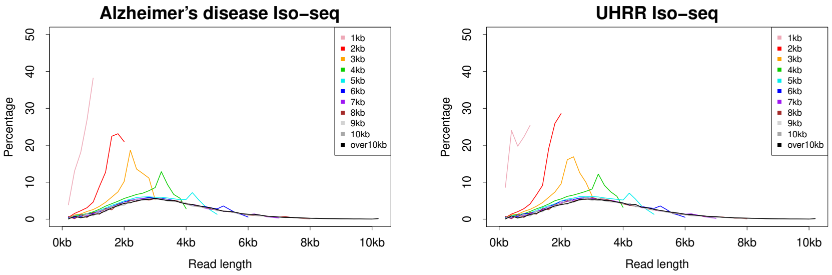

Figure S13: Read length distribution of PacBio Iso-seq (HiFi reads). Reads were grouped by 1 kb by their template length. Each graph shows the distribution of the read length, where colors of plotted lines represent read groups (e.g., '1kb' refers to a read group with their template accuracy of 1–1000 bp). CLR reads were simulated using the PBSIM3 quality score models, and HiFi reads were generated by ccs software as consensus sequences from PBSIM3 outputs. The template transcript from which each read was most likely sequenced was obtained from alignments between the reads and their reference genomes.

(A) Real reads

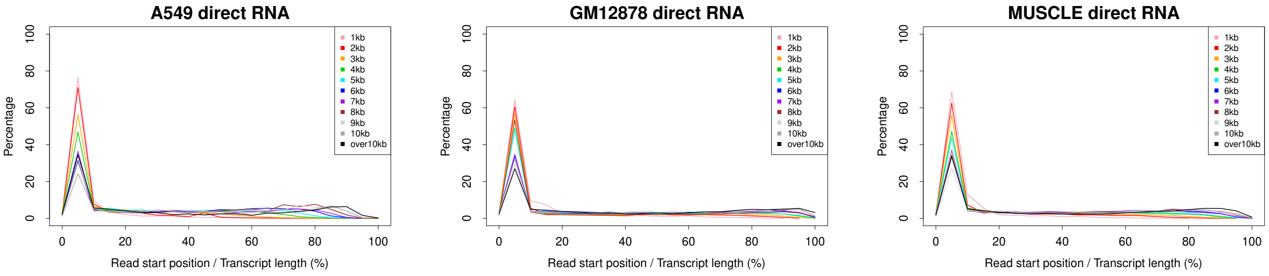

(B) Simulated reads

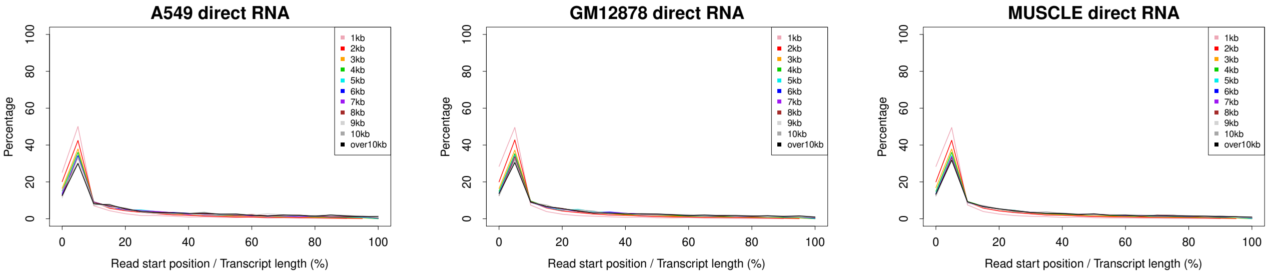

Figure S14: Read start positions of ONT direct RNA on their template transcripts. Reads were grouped by 1 kb by their template length. Each graph shows the distribution of the read start positions, where colors of plotted lines represent read groups (e.g., '1kb' refers to a read group with their template length of 1–1000 bp). The horizontal axis indicates the position of the read start positions in the total length of their templates, which was calculated by dividing the read start position by the total length of the template; the graph is plotted in 5% increments, with the left edge of the graph showing the percentage of reads starting exactly at the 5'end of the template. ONT direct RNA were simulated using the PBSIM3 quality score models. The template transcript from which each read was most likely sequenced and the read start position was obtained from alignments between the reads and their reference transcriptomes.

(A) Real reads

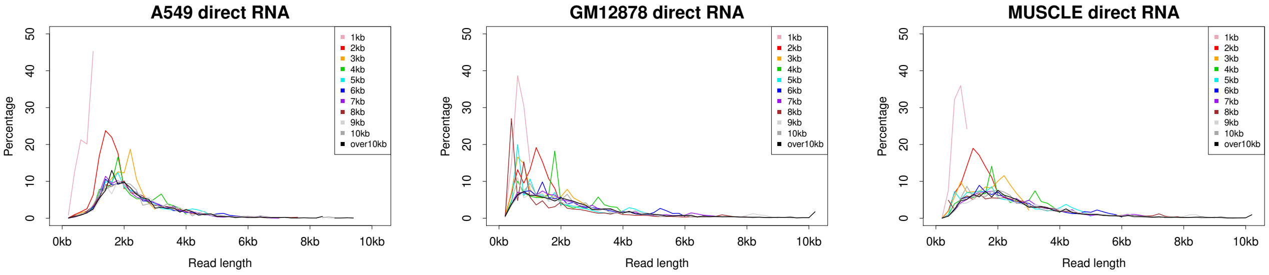

(B) Simulated reads

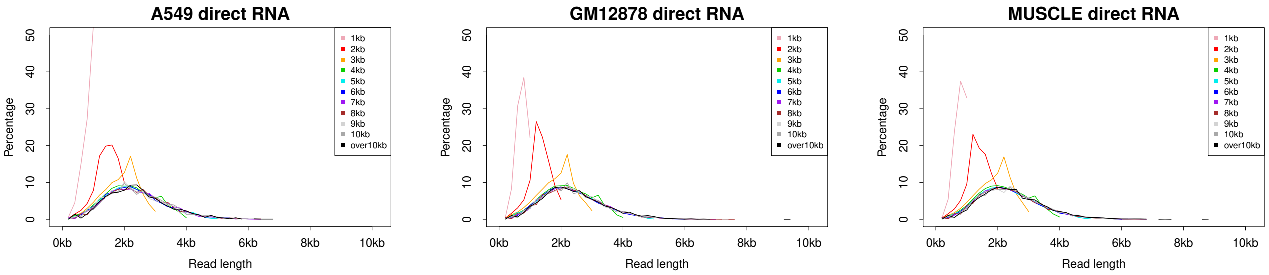

Figure S15: Read length distribution of ONT direct RNA. Reads were grouped by 1 kb by their template length. Each graph shows the distribution of the read length, where colors of plotted lines represent read groups (e.g., '1kb' refers to a read group with their template accuracy of 1–1000 bp). ONT direct RNA were simulated using the PBSIM3 quality score models. The template transcript from which each read was most likely sequenced was obtained from alignments between the reads and their reference genomes.

(A) Real reads

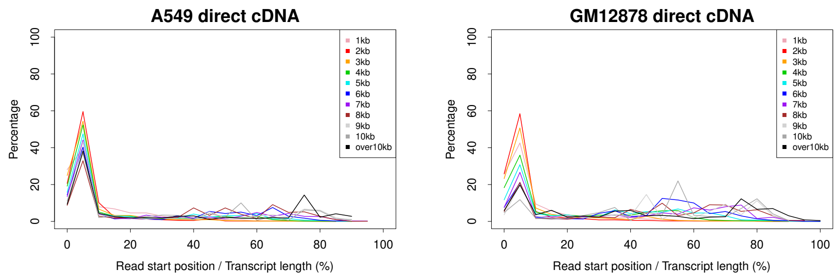

(B) Simulated reads

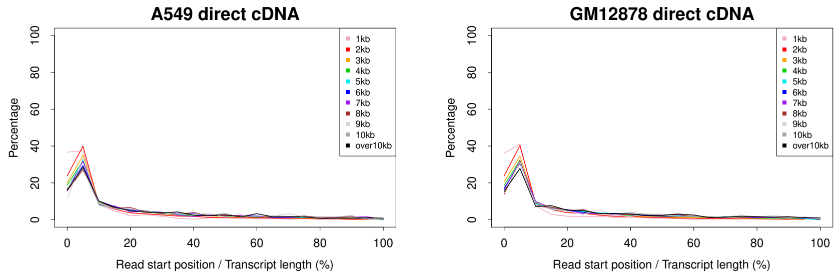

Figure S16: Read start positions of ONT direct cDNA on their template transcripts. Reads were grouped by 1 kb by their template length. Each graph shows the distribution of the read start positions, where colors of plotted lines represent read groups (e.g., '1kb' refers to a read group with their template length of 1–1000 bp). The horizontal axis indicates the position of the read start positions in the total length of their templates, which was calculated by dividing the read start position by the total length of the template; the graph is plotted in 5% increments, with the left edge of the graph showing the percentage of reads starting exactly at the 5'end of the template. ONT direct cDNA were simulated using the PBSIM3 quality score models. The template transcript from which each read was most likely sequenced and the read start position was obtained from alignments between the reads and their reference transcriptomes.

(A) Real reads

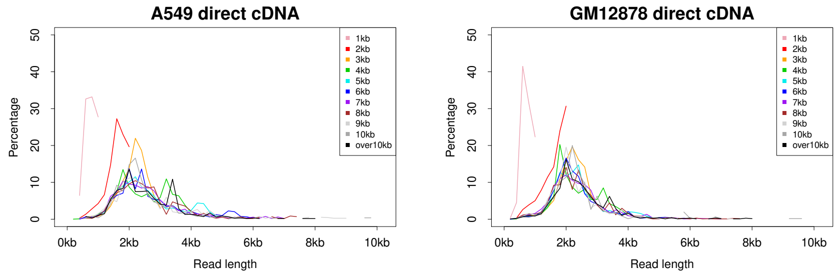

(B) Simulated reads

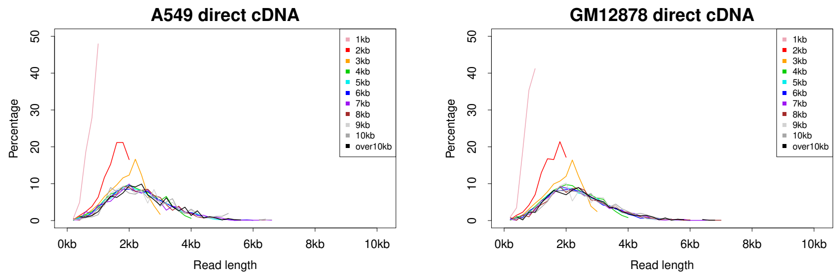

Figure S17: Read length distribution of ONT direct cDNA. Reads were grouped by 1 kb by their template length. Each graph shows the distribution of the read length, where colors of plotted lines represent read groups (e.g., '1kb' refers to a read group with their template accuracy of 1–1000 bp). ONT direct cDNA were simulated using the PBSIM3 quality score models. The template transcript from which each read was most likely sequenced was obtained from alignments between the reads and their reference genomes.
